# Regulation of Mitophagy by Low-Intensity Pulsed Ultrasound Attenuates Endothelial Dysfunction

**DOI:** 10.3390/metabo16050329

**Published:** 2026-05-15

**Authors:** Yucong Shi, Baotian Zhao, Yuhong Wei, Dongxu Lu, Haixia Liu, Yinzhu Chu

**Affiliations:** 1 The First Clinical Medical College of Harbin Medical University, Harbin 150001, China; 2023020734@hrbmu.edu.cn (Y.S.); 2024021131@hrbmu.edu.cn (B.Z.); 2023020763@hrbmu.edu.cn (Y.W.); ludongxu@hrbmu.edu.cn (D.L.); 2 NHC Key Laboratory of Cell Transplantation, Harbin 150001, China; 3 Heilongjiang Provincial Key Laboratory of Hepatosplenic Surgery, Harbin 150001, China; 4 The Third Clinical College of Harbin Medical University, Harbin 150081, China; 900116@hrbmu.edu.cn

**Keywords:** low-intensity pulsed ultrasound, mitophagy, endothelial cells, type 2 diabetes mellitus, high glucose

## Abstract

**Background**: Diabetic vascular complications are a major cause of poor prognosis in patients with diabetes mellitus (DM). Mitophagy activation is a potential therapeutic target for type 2 diabetes mellitus (T2DM), but the role of low-intensity pulsed ultrasound (LIPUS) in this context remains unclear. **Methods**: The biological effects of LIPUS on endothelial cells under high glucose conditions were systematically evaluated using high glucose-treated human umbilical vein endothelial cells (HUVECs) and aortic tissues from diabetic rats as models, in combination with bioinformatics analysis and standard molecular and cellular biology techniques. Histological staining was further used to assess the protective role of LIPUS in the aortas of diabetic rats. **Results**: Bioinformatics analysis predicted that high glucose induces mitochondrial dysfunction, suppresses autophagy in HUVECs, impairs endothelial cell function, and activates fibroblasts. In vitro results were in agreement with these predictions. LIPUS treatment significantly counteracted these effects, restoring migration (*p* < 0.001) and angiogenesis (*p* < 0.05), increasing proliferation (*p* < 0.001), and decreasing apoptosis (*p* < 0.05). Mechanistically, LIPUS enhanced mitophagy, and its therapeutic effects were markedly diminished upon addition of the autophagy inhibitor 3-Methyladenine (3-MA). In vivo, LIPUS attenuated aortic endothelial damage and reduced collagen deposition in diabetic rats (*p* < 0.01). **Conclusions**: LIPUS may ameliorate hyperglycemia-induced endothelial cell dysfunction by activating mitophagy, and it also attenuates pathological damage in the abdominal aorta of diabetic rats, thereby providing experimental evidence for its application in the treatment of diabetic macrovascular complications.

## 1. Introduction

Diabetes mellitus is a serious ongoing metabolic disorder caused by either the pancreas not producing enough insulin or the body’s inability to use insulin effectively [1]. With ongoing social development, the number of diabetic patients continues to rise. According to the 11th edition of the Global Diabetes Map published by the International Diabetes Federation, diabetes-related deaths exceeded 3.4 million in 2024, accounting for 9.3% of all global deaths [2]. A hallmark of diabetes is persistent hyperglycemia accompanied by vascular complications [3]. A persistent hyperglycemic state leads to damage to the cardiovascular system [4], posing a serious threat to patient health and survival. The endothelial cells of diabetic patients are vulnerable to injury and are particularly susceptible to atherosclerosis, leading to vascular complications [5]. Current management of diabetic vascular complications primarily relies on pharmacologic therapies for symptom control, and these have not substantially reduced disease prevalence. Despite the emergence of novel interventions, numerous unknowns remain before their clinical translation, and their long-term safety in humans requires further evaluation. The development of safe and effective non-pharmacological intervention strategies is therefore of significant clinical value.

High glucose induces mitochondrial damage, and damaged mitochondria produce excessive superoxide, leading to endothelial dysfunction [6,7]. Mitophagy selectively removes damaged mitochondria, thereby helping to maintain mitochondrial integrity and support cellular homeostasis [8]. Impaired mitophagy is considered to be involved in the pathogenesis of type 2 diabetes mellitus (T2DM) [9]. High glucose not only induces mitochondrial damage in endothelial cells but also impairs mitophagy [10], leading to the accumulation of damaged mitochondria and excessive production of reactive oxygen species, which further exacerbates cellular injury. Previous studies suggest that restoring mitophagy may represent a reliable and effective therapeutic target for T2DM [11]. However, for diabetic macrovascular complications, whether or not activating mitophagy in vascular endothelial cells can counteract endothelial dysfunction remains poorly explored.

Low-intensity pulsed ultrasound (LIPUS), a non-invasive therapeutic modality whose safety is already documented [12], delivers moderate-frequency, low-intensity ultrasound waves in a pulsed mode, with energy levels well below 3 W/cm^2^. This approach significantly reduces thermal effects [13] while optimizing mechanical impacts, primarily through cavitation and mechanical actuation [14]. As a therapeutic ultrasound technology, LIPUS effectively initiates biological processes, including gene expression, cellular signaling, enzyme activity, cell proliferation, and differentiation [15,16,17]. LIPUS also plays an important role in promoting fracture healing, wound repair, immune regulation, inflammation reduction, and increased capillary permeability [18]. Many studies in cardiovascular research have utilized the cavitation effect of LIPUS for nanodrug delivery without thoroughly investigating its mechanisms [19]. LIPUS has been shown to inhibit oxidative stress-induced endothelial injury [20]. Clinical studies have also confirmed that LIPUS improves clinical symptoms, perfusion parameters, and endothelial function in patients with vascular diseases; however, these findings remain at a macroscopic level, without the investigation of their underlying mechanisms [21,22]. Guo et al. [23] demonstrated that therapeutic ultrasound induces mitophagy in β-cells. Chen et al. [24] provided evidence that LIPUS activates mitophagy in damaged corpus cavernosum endothelial cells. Nevertheless, despite the growing body of evidence, the role of LIPUS in diabetic macrovascular complications—particularly whether or not LIPUS activates mitophagy in endothelial cells to alleviate macrovascular injury—remains largely underexplored.

Based on this, this study focuses on endothelial cell dysfunction, a common pathological feature of diabetes and its associated macrovascular complications. Combining the potential advantages of LIPUS as a non-invasive physical intervention, we further investigated whether LIPUS ameliorates high glucose-induced endothelial injury by activating mitophagy. This study provides experimental evidence for the development of novel adjunctive therapeutic strategies for diabetic macrovascular complications and establishes a theoretical foundation for expanding the clinical application of LIPUS.

## 2. Materials and Methods

### 2.1. Cell Culture and Treatment

RRID: CL-0675 human umbilical vein endothelial cells (HUVECs) were acquired from Wuhan Procell Biotechnology Co., Ltd. (Wuhan, China). The cell line has been authenticated by STR profiling. The cells were cultured in ECM medium (ScienCell, San Diego, CA, USA). The cells were maintained at 37 °C in 5% CO_2_ atmosphere.

Cells seeded in 6-well plates were randomly divided into three groups: negative control (NC), high glucose (HG), and high glucose with low-intensity pulsed ultrasound treatment (HG + LIPUS). Cells in the HG and HG + LIPUS groups were exposed to 30 mM high glucose [25] for 24 h to establish a hyperglycemic injury model. In the HG + LIPUS group, following modeling, a layer of ultrasound coupling gel, 2 mm thick, was spread on the base of the culture plate to minimize ultrasound signal attenuation due to the presence of air. Treatment was then administered with a Nu-Tek UT1021 low-intensity pulsed ultrasound device (Nu-Tek, Shenzhen, China). Before each daily treatment, the culture medium was replaced with fresh high-glucose medium (cells in the NC and HG groups also received fresh medium every day). The parameters of LIPUS treatment applied were as follows: pulse repetition frequency = 100 Hz; center frequency = 1 MHz; spatial peak temporal average intensity = 100 mW/cm^2^; acoustic output power = 0.5 W; duty cycle = 20%, 20 min/day for 7 consecutive days, whereas the NC and HG groups underwent the same procedures without the LIPUS therapy.

### 2.2. Animal Models and Therapy

Twenty 5-week-old male Sprague Dawley (SD) rats were purchased from SiPeiFu (Beijing) Biotechnology Co., Ltd., Beijing, China, [License No.: SCXK(Jing) 2024-0001]. The animals were housed under controlled temperature (22–24 °C), and relative humidity was maintained at 40–60%, with a 12 h light and dark cycle and unlimited access to food and water. Following a one-week adaptation, experiments were initiated. All procedures received approval from the Laboratory Animal Management and Ethics Review Committee of First Affiliated Hospital of Harbin Medical University (No.: 2024085).

Six-week-old rats were used for subsequent experiments. They were divided into two groups at random: Control group (*n* = 4) and Model group (*n* = 16). The Control group received standard chow for four weeks. The Model group was fed a high-fat diet for four weeks and then fasted overnight. Streptozotocin (STZ) (Solarbio, Beijing, China) was dissolved in 10 mM sodium citrate buffer (pH = 4.5) and administered as a single intraperitoneal injection at 35 mg/kg. After fasting, rats in the Control group received an equivalent volume of sodium citrate buffer via intraperitoneal injection. Successful model establishment was confirmed when random blood glucose levels measured via the tail vein exceeded 16.0 mmol/L for three consecutive days, starting 72 h after STZ injection [26]. Among the Model group, a total of eight rats met the criteria and were then randomly divided into two groups: diabetes mellitus (DM, *n* = 4) and diabetes mellitus with low-intensity pulsed ultrasound (DM + LIPUS, *n* = 4). Rats in the DM + LIPUS group received LIPUS treatment. Following induction of anesthesia with isoflurane, rats were secured in a supine position. The abdominal area was shaved and cleansed, after which ultrasound coupling gel was applied to facilitate the ultrasound intervention. The same parameters as those in the in vitro experiments were used: pulse repetition frequency = 100 Hz; center frequency = 1 MHz; spatial peak temporal average intensity = 100 mW/cm^2^; acoustic output power = 0.5 W; duty cycle = 20%, 20 min/day for 7 consecutive days.

### 2.3. Bioinformatics Analysis

The dataset GSE241565 [27] was acquired from the Gene Expression Omnibus (GEO, https://www.ncbi.nlm.nih.gov/geo/, accessed on 28 April 2026). The R package DESeq2 [28] was utilized to conduct differential expression analysis between normal samples and HUVECs treated with 30 mM glucose, which is designed for differential analysis of count data. Differentially expressed genes (DEGs) were identified as having a *p*-value < 0.05 and |fold change (FC)| > 1.2. DEGs; those with FC > 1.2 were considered upregulated, and those with FC < −1.2 were considered downregulated. Functional enrichment analyses were conducted, including the Kyoto Encyclopedia of Genes and Genomes (KEGG) pathway, Gene Ontology (GO), and Gene Set Enrichment Analysis (GSEA). The GO analysis covered biological process (BP) and cellular component (CC) categories. Terms with *p* < 0.05 were regarded as statistically significant. Results were visualized via the online platform Xiantao Academic (https://www.xiantaozi.com). For GSEA, gene sets “c2.all.v2022.1.Hs.symbols” and “c5.all.v2022.1.Hs.symbols” were used, and corresponding GSEA plots and ridgeline plots were generated.

### 2.4. Wound Healing Assay

HUVECs were seeded into 6-well plates at 1 × 10^6^ cells per well and cultured in complete medium. After 48 h, when the cells became fully confluent, a 200 μL pipette tip was used to make a linear wound. Detached cells were gently washed away with PBS, and serum-free basal medium was added. Images of the same wound region were captured at 0, 12, and 24 h with a Zeiss Axio Observer.A1 inverted microscope (ZEISS, Oberkochen, Germany). ImageJ software 2.16.0/1.54p (National Institutes of Health, Bethesda, MD, USA) was used to quantify the migration area in relation to the total scratch area.

### 2.5. Transwell Migration Assay

Cells underwent a 12 h serum starvation beforehand. Afterward, cells were seeded at a density of 5 × 10^4^ cells/200 μL into the upper chamber of the Transwell system. The lower chamber was filled with complete medium, submerging the bottom of the insert. The upper chamber was removed after 24 h, cleaned with PBS, immersed in 4% paraformaldehyde (PFA) for 15 min, and colored with 0.1% crystal violet (Beyotime, Shanghai, China) for 15 min. Migrated cells were captured with a Zeiss Axio Observer.A1 inverted microscope and counted with ImageJ software.

### 2.6. Tube Formation Assay

Matrix-Gel™ Basement Membrane Matrix (Mogengel-Bio, Xiamen, China), in a volume of 20 μL, was used to precoat a 24-well plate. HUVECs, previously serum-starved for 12 h, were seeded at 1.5 × 10^5^ cells per well onto the gel. After 6 h, tubular structures were photographed with a Zeiss Axio Observer.A1 inverted microscope. ImageJ software was used to evaluate tube formation.

### 2.7. Spheroid Sprouting Assay

A methylcellulose stock solution (1.2% *w*/*v*) was prepared in ECM medium. The cell concentration was adjusted to 2 × 10^4^/mL to obtain a single-cell suspension. A 4 mL aliquot of this suspension was mixed with 1 mL of the methylcellulose stock solution, and the combined solution was gently mixed. Subsequently, 40 μL of the mixture was inoculated onto the lid of a 60 mm culture dish. Twenty drops were placed on each lid. The lid was then carefully inverted to form a hanging drop and incubated in a 37 °C incubator with 5% CO_2_ for 24 h to allow spheroid formation. The resulting spheroids were collected and subjected to low-speed centrifugation, after which the supernatant was discarded. The spheroids were resuspended in a methylcellulose–collagen medium supplemented with 20% FBS and seeded into a 24-well plate. The plate was incubated in a CO_2_ incubator for 24 to 48 h. The images were photographed with a Zeiss Axio Observer.A1 inverted microscope.

### 2.8. EdU Assay

Cells were seeded into a 48-well plate at a concentration of 1 × 10^4^ cells per well and incubated overnight. Cell proliferation ability was evaluated by the EdU Cell Proliferation Kit (Beyotime, Shanghai, China). The increased cells were marked according to the manufacturer’s instructions. Hoechst 33342 (Beyotime, Shanghai, China) stained the nuclei. Images were observed and captured under an EVOS M5000 fluorescence microscope (Thermo Fisher Scientific, Waltham, MA, USA), and EdU-positive cells were counted with ImageJ software.

### 2.9. TUNEL Assay

A 48-well plate was used to seed the cells. After adhesion, the samples were fixed in 4% PFA for 30 min and then permeabilized in 0.3% Triton X-100 (Solarbio, Beijing, China) for 5 min. Apoptotic cells were identified by the TUNEL Apoptosis Assay Kit (Beyotime, Shanghai, China), according to the manufacturer’s instructions. Hoechst 33342 was used to stain the nuclei for 10 min in the dark. TUNEL-positive cells were imaged by an EVOS M5000 fluorescence microscope and quantified with ImageJ software.

### 2.10. Western Blot

Cells were lysed in RIPA (Beyotime, Shanghai, China) buffer that included protease and phosphatase inhibitors (Solarbio, Beijing, China). Protein levels were measured with a BCA protein assay kit (Beyotime, Shanghai, China), and samples were normalized to equal concentrations. We used 12.5% SDS-PAGE to separate the proteins and then transferred them to PVDF membranes. The membrane was then blocked for 60 min at room temperature with 5% BSA (Solarbio, Beijing, China) blocking buffer, combined with the following primary antibodies: Rabbit anti-p62 (1:8000, Abmart, Shanghai, China, T55546), anti-LC3 (1:1000, Abmart, T55992), anti-TOM20 (1:1000, Abmart, T55527), anti-Bcl-2 (1:1000, Abmart, T40056), and anti-Bax (1:1000, Abmart, T40051) antibodies. Next, it was exposed to a secondary antibody, Mouse anti-β-actin antibody (1:10,000, Abmart, T40104), for 60 min in a room-temperature environment. Blots were developed with a supersensitive ECL chemiluminescence kit (MCE, Shanghai, China) in a ChampChemi TM 910 chemiluminescent imaging system (Sage Creation, Beijing, China). The band intensities were quantified by ImageJ software, and the expression levels of target proteins were normalized to β-actin.

### 2.11. Immunofluorescence

Sterile coverslips were placed in a 24-well plate, and cells were seeded at 5 × 10^4^ cells per well. After treatment, cells were treated with 4% PFA for 10 min, permeabilized with 0.1% Triton X-100 for 5 min, and blocked with 5% BSA for 60 min. The primary antibody, Rabbit anti-LC3 antibody (1:200, Abmart, T55992) and mouse anti-TOM20 antibody (1:200, Proteintech, Wuhan, China, 666777-1-Ig), diluted in blocking solution, was applied overnight at 4 °C. Incubation of the secondary antibody occurred for 60 min at room temperature in the dark, and the nuclei were stained with DAPI (Beyotime, Shanghai, China) for 10 min without light. Images were acquired with an EVOS M5000 fluorescence microscope, and fluorescence intensity and colocalization were analyzed with ImageJ software.

### 2.12. Histological Staining

After treatment, rats were euthanized under isoflurane anesthesia. The abdominal aorta was excised, rinsed, and preserved in 4% PFA. Tissue samples were embedded in paraffin. A HistoCore AUTOCUT automated rotary microtome (Leica Biosystems, Nussloch, Germany) was used to section the tissue into 5 μm slices. Sections were stained with H&E (Solarbio, Beijing, China), modified Sirius red (Solarbio, Beijing, China), and Masson’s trichrome staining kits (Servicebio, Wuhan, China). Upon completion of dehydration, clearing, and mounting, the slides were digitally scanned with an Olympus VS200 whole-slide scanner (Olympus Corporation, Tokyo, Japan). The collagen fiber staining results of the vessel walls were quantified via ImageJ software.

### 2.13. Statistical Analysis

Data analysis was performed with GraphPad Prism 10.1.2 (GraphPad Software, Inc., San Diego, CA, USA). All data are presented as mean ± standard deviation (SD). Normality of distribution was assessed by the Shapiro–Wilk test. An unpaired two-tailed Student’s *t*-test was used for two-group comparison. Multiple group comparisons were performed by one-way ANOVA, followed by Dunnett‘s post hoc test. Statistical significance was assigned to *p*-value < 0.05, with each experiment being independently repeated at least three times.

## 3. Results

### 3.1. Analysis of Differential Gene Expression and Functional Enrichment

**Figure 1:**
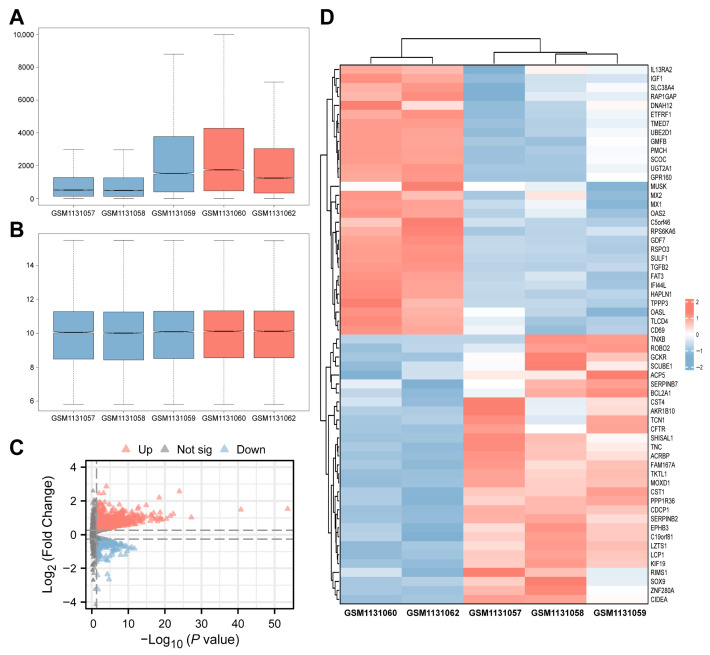
Transcriptomic alterations in human umbilical vein endothelial cells (HUVECs) induced by high glucose. (**A**) Boxplot of gene expression data before normalization. (**B**) Boxplot of gene expression data after normalization. (**C**) Volcano plot of differentially expressed genes (DEGs) between the Control group and the high glucose (HG) group. Red triangles represent upregulated genes, and blue triangles represent downregulated genes. (**D**) Heatmap of key DEGs in high-glucose and Control groups. Rows represent individual genes; columns represent samples. The color scale indicates relative expression levels (red: upregulated, blue: downregulated).

To explore the impact of high glucose on HUVECs function and the underlying mechanisms thereof, RNA-Seq data (GSE241565) were sourced from the GEO database, including three normal (GSM7731057, GSM7731058, GSM7731059) and two high-glucose-treated HUVECs samples (GSM7731060, GSM7731062) (raw data for the GSM7731061 dataset were not uploaded). Expression levels were normalized across all samples to facilitate subsequent analysis (Figure 1A,B). A total of 2881 DEGs were identified, with 2021 upregulated and 860 downregulated (Figure 1C). A heatmap of the top 30 upregulated and downregulated genes was generated with the ComplexHeatmap package (Figure 1D), showing different expression patterns between the high-glucose and normal groups.

**Figure 2:**
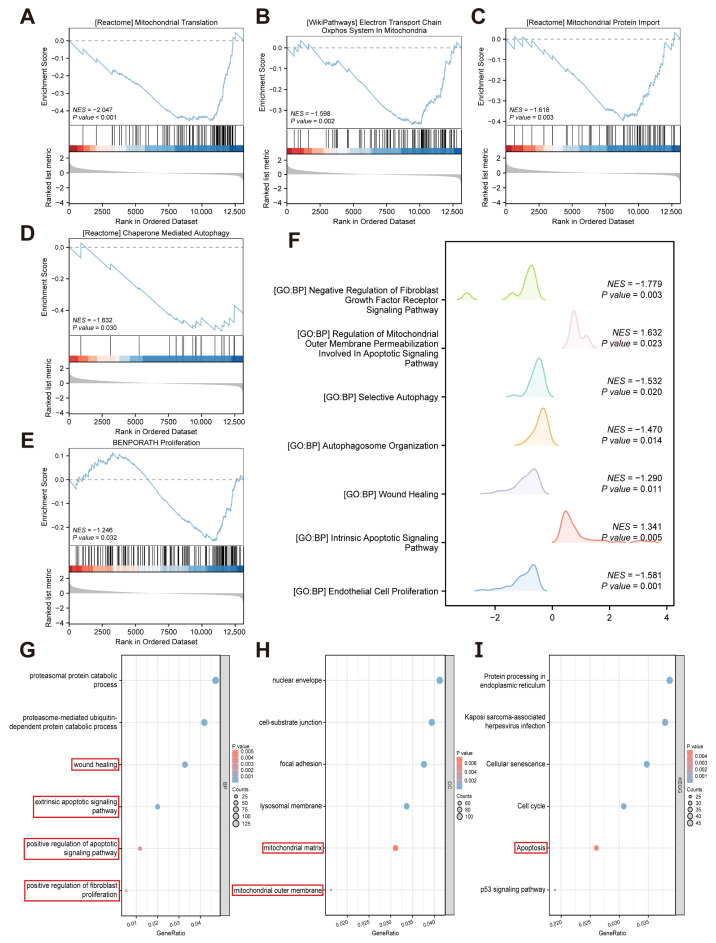
Gene Set Enrichment Analysis (GSEA), Gene Ontology (GO), and Kyoto Encyclopedia of Genes and Genomes (KEGG) enrichment analyses of HUVECs in control and HG groups. (**A**–**E**) GSEA plots of representative gene sets (c2.all.v2022.1.Hs.symbols). Blue curve: enrichment score (ES); black bars: gene hits; normalized enrichment score (NES) and *p*-values as indicated in the plots. Red: up-regulated genes, blue: down-regulated genes. (**Bottom panel**): Ranked list metric for all genes. (**F**) Ridgeline plot of representative gene sets (c5.all.v2022.1.Hs.symbols). X-axis: enrichment score distribution; y-axis: enriched gene set names. The NES and *p*-values are as indicated in the plot. (**G**,**H**) GO enrichment bubble plots for (**G**) biological process (BP) and (**H**) cellular component (CC) categories. (**I**) Bubble plot showing KEGG pathway enrichment results. In the bubble plots, the bubble size corresponds to the number of genes enriched in a term, while the color gradient (red to blue) signifies the *p*-value (from high to low). Key functional entries and pathways of interest are highlighted with red boxes.

Based on the distinct expression patterns observed between the high-glucose and Control groups, we further explored potential biological mechanisms and pathways. GSEA based on the c2 gene set showed that gene sets related to mitochondria, autophagy, and proliferation were significantly downregulated under high glucose conditions (Figure 2A–E). GSEA analysis based on the c5 gene set showed that gene sets related to apoptosis were significantly upregulated under high glucose conditions, while gene sets related to the negative regulation of the fibroblast growth factor receptor signaling pathway, autophagy, wound healing, and endothelial cell proliferation were significantly downregulated under high glucose conditions (Figure 2F). Some of the enriched terms for BP and CC are shown in Figure 2G,H. In BP, DEGs were enriched in regards to wound healing, extrinsic apoptotic signaling pathway, positive regulation of apoptotic signaling pathway, and positive regulation of fibroblast proliferation. For CC, DEGs were enriched in the mitochondrial matrix and mitochondrial outer membrane. KEGG analysis further indicated DEG enrichment in the apoptosis pathway (Figure 2I). Collectively, these bioinformatics results suggest that under high-glucose conditions, HUVECs display mitochondrial dysfunction, suppressed autophagy level, and impaired proliferation and wound healing capacity, along with enhanced fibroblast proliferation.

### 3.2. LIPUS Rescues the High Glucose-Induced Angiogenesis Impairment in HUVECs

Endothelial cell migration and tube formation are crucial components of angiogenesis. The influence of LIPUS on HUVECs angiogenesis in a hyperglycemic setting was evaluated via wound healing, Transwell, tube formation, and endothelial cell spheroid sprouting assays.

**Figure 3:**
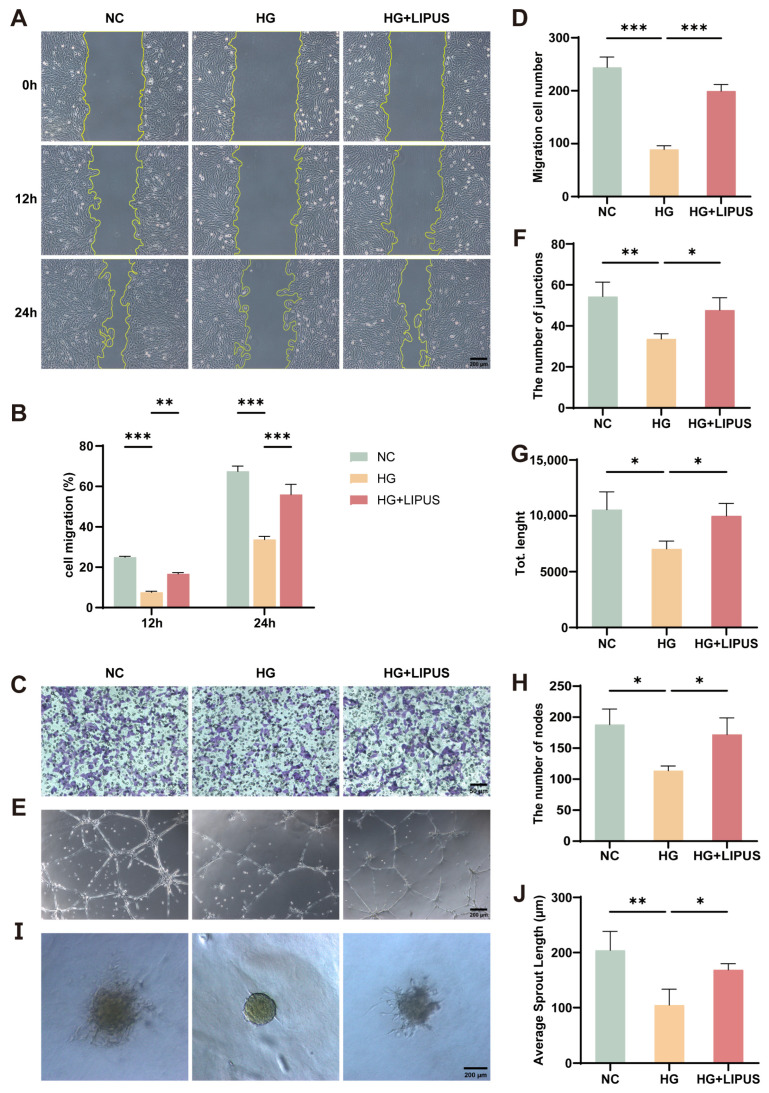
Low-intensity pulsed ultrasound (LIPUS) rescues the damage to the angiogenic potential of HUVECs caused by high glucose. (**A**,**B**) Representative images and statistical graph of wound healing assay results for HUVECs in each group after 12 and 24 h; two-way ANOVA. The scale bar was set to 200 μm (*n* = 3). (**C**,**D**) Representative images and statistical graph of Transwell migration results for HUVECs in each group; one-way ANOVA. The scale bar was set to 50 μm (*n* = 3). (**E**–**H**) Angiogenic potential of HUVECs treated with NC, HG, and HG + LIPUS was analyzed by tube formation assay and quantified with (**F**) the number of junctions, (**G**) total length, and (**H**) the number of nodes; one-way ANOVA. The scale bar was set to 200 μm (*n* = 3). (**I**,**J**) Angiogenic potential of HUVECs treated with NC, HG, and HG + LIPUS was analyzed by endothelial cell spheroid sprouting assay and quantified with average sprout length; one-way ANOVA. The scale bar was set to 200 μm (*n* = 3). * *p* < 0.05, ** *p* < 0.01, *** *p* < 0.001. Error bars represent standard deviation (SD) of the mean.

The wound healing assay results showed that compared with the NC group, the cell migration rate in the HG group was significantly reduced at both 12 h and 24 h. After LIPUS intervention (HG + LIPUS group), the migration rate was significantly higher than that in the HG group (Figure 3A,B). The Transwell assay results were consistent with these findings, as the number of migrated cells in the HG + LIPUS group was significantly greater than that in the HG group (Figure 3C,D). These results indicate that high glucose inhibits the migration ability of HUVECs, and LIPUS counteracts this effect.

The tube formation ability was evaluated by quantifying the number of junctions, total tube length, and number of nodes in each group. We further assessed the sprouting potential of spheroids using the average sprout length. The results showed that the tube formation capacity and sprouting potential of spheroids in the HG group were decreased compared with those of the NC group, whereas LIPUS significantly restored them (Figure 3E–J). Together, this information shows that LIPUS rescues the high glucose-induced deficit in the angiogenic potential of HUVECs.

### 3.3. LIPUS Enhances Proliferation and Suppresses Apoptosis in High Glucose-Exposed HUVECs

**Figure 4:**
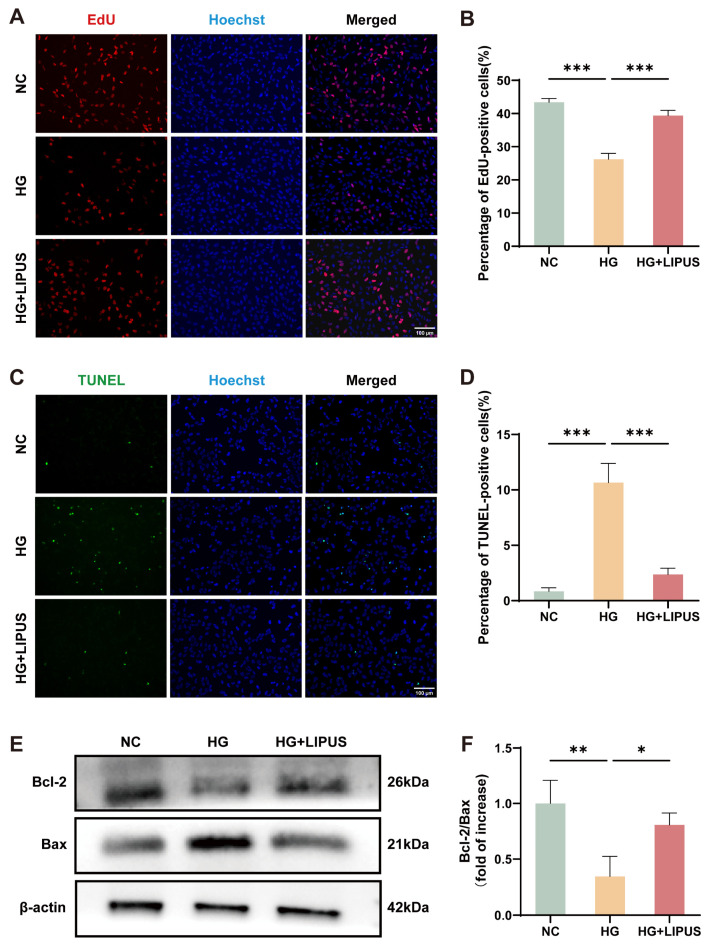
LIPUS promotes proliferation and inhibits apoptosis of HUVECs under high glucose conditions. (**A**,**B**) Fluorescence images and statistical analysis of EdU-positive cells in HUVECs after high glucose stimulation and LIPUS treatment; EdU-positive cells (red)/DAPI (blue); one-way ANOVA. The scale bar was set to 100 μm (*n* = 3). (**C**,**D**) Fluorescence images and statistical analysis of TUNEL-positive cells in HUVECs after high-glucose stimulation and LIPUS treatment; TUNEL-positive cells (green)/DAPI (blue); one-way ANOVA. The scale bar was set to 100 μm (*n* = 3). (**E**,**F**) Representative protein bands and quantitative analysis of Bcl-2 and Bax protein expression levels; one-way ANOVA (*n* = 3); * *p* < 0.05, ** *p* < 0.01 *** *p* < 0.001. Error bars represent SD of the mean.

To assess the role of LIPUS in endothelial cell proliferation and apoptosis under hyperglycemic conditions, EdU and TUNEL assays were performed. A significant suppression of proliferation was observed in the HG group relative to the NC group, based on EdU analysis, whereas LIPUS treatment increased cell proliferation under high glucose conditions (Figure 4A,B). Relative to the NC group, TUNEL staining indicated elevated apoptosis in the HG group, which was attenuated by LIPUS (Figure 4C,D). The Bcl-2 protein family plays a critical role in regulating the mitochondrial-mediated apoptotic pathway [29,30]. Western blot analysis demonstrated that the Bcl-2/Bax ratio was significantly decreased in the HG group compared to that in the NC group. However, this reduction was significantly attenuated in the HG + LIPUS group. (Figure 4E,F). The above results suggest that high glucose suppresses proliferation and promotes apoptosis in HUVECs and that LIPUS counteracts both of these adverse effects.

### 3.4. LIPUS Can Activate Mitophagy in HUVECs Under High Glucose Conditions

**Figure 5:**
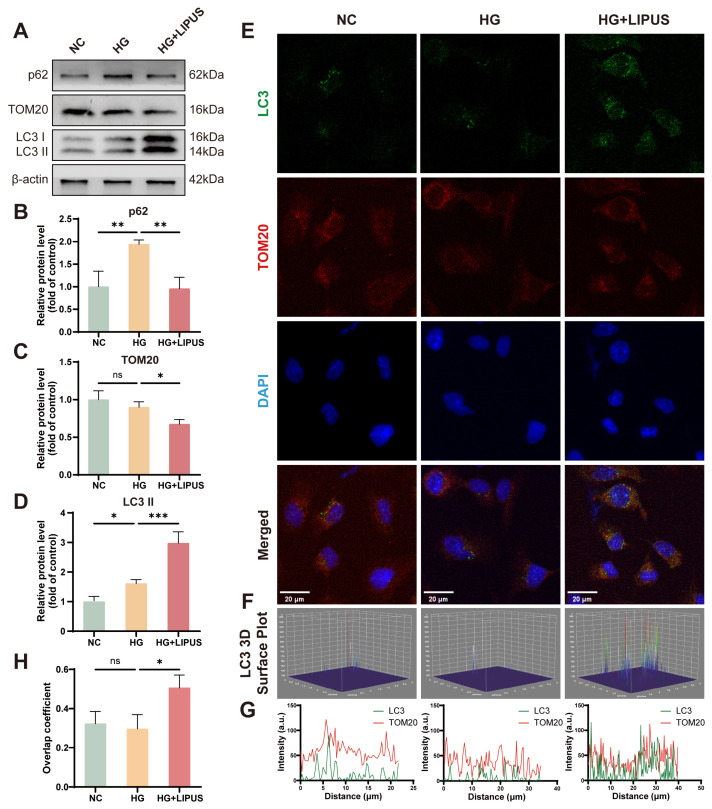
LIPUS promotes mitophagy in high glucose-stimulated HUVECs. (**A**–**D**) Representative protein bands and quantitative analysis of p62, LC3-II, and TOM20 protein levels; one-way ANOVA (*n* = 3). (**E**) Immunofluorescence co-staining of LC3 (green), TOM20 (red), and DAPI (blue). The scale bar was set to 20 μm (*n* = 3). (**F**) 3D surface plot of LC3 fluorescence. (**G**) Fluorescence intensity distribution profiles for LC3 and TOM20. Green represents LC3; red represents TOM20. (**H**) Quantification of LC3-TOM20 co-localization. Co-localization was quantified with the overlap coefficient calculated with ImageJ (Colocalization Finder plugin). A total of 16–21 cells per group were analyzed; one-way ANOVA (*n* = 3); ns, not significant; * *p* < 0.05, ** *p* < 0.01, *** *p* < 0.001. Error bars represent SD of the mean.

Mitophagy was first evaluated by Western blotting with the following protein markers: the autophagic flux indicators p62 and LC3-II and the mitochondrial outer membrane protein TOM20. LC3, a widely used autophagic marker [31], and TOM20, the expression level of which is associated with mitochondrial mass [32], are frequently employed to monitor autophagic activity and mitochondrial content, respectively. p62 is a major cargo receptor for selective autophagy and accumulates in cells when autophagy is inhibited [33,34]. In comparison to that in the HG group, LIPUS significantly reduced p62 and TOM20 expression, while increasing LC3-II levels (Figure 5A–D). The enhanced LC3-TOM20 co-localization, reflecting the recruitment of autophagosomes to mitochondria, is a key indicator of activated mitophagy for eliminating impaired mitochondria. The LC3-TOM20 co-localization was then analyzed by immunofluorescence, and the results are shown in Figure 5F–H. The results showed that compared with the results for HG group, the LC3-TOM20 co-localization coefficient increased after LIPUS treatment (Figure 5G,H). In addition, both Western blot (Supplementary Figure S1A–D) and immunofluorescence (Supplementary Figure S1E–G) results showed that LIPUS treatment alone had no effect on mitophagy in HUVECs, indicating that LIPUS does not non-specifically activate mitophagy under physiological conditions. Taken together, these results suggest that LIPUS can activate mitophagy in HUVECs under high-glucose conditions.

### 3.5. Mitophagy Inhibition Can Weaken the Beneficial Effects of LIPUS

**Figure 6:**
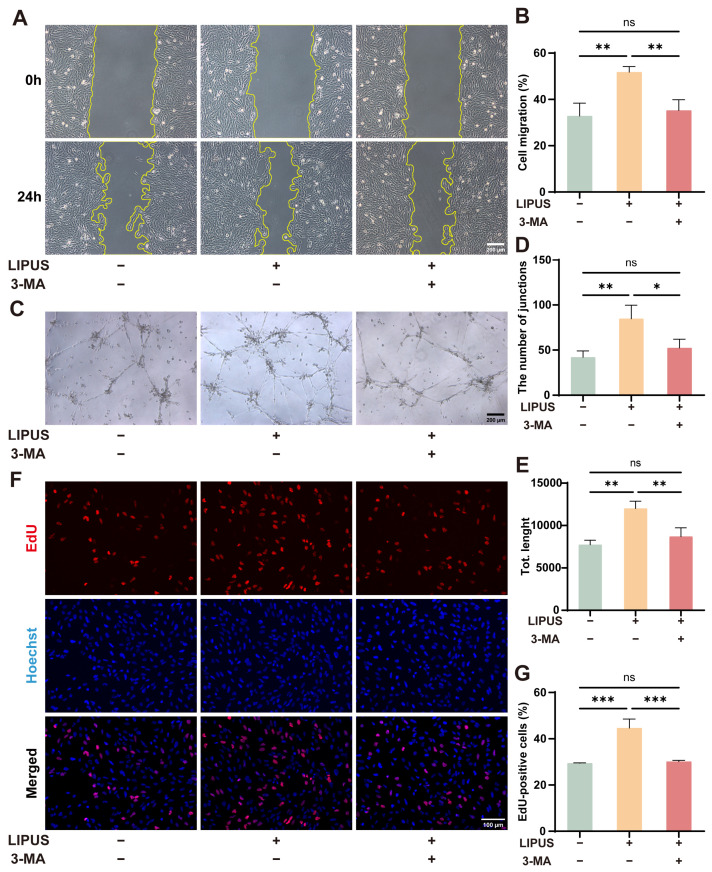
Mitophagy inhibition can weaken the beneficial effects of LIPUS. (**A**,**B**) Results of wound healing assay at 24 h in HG + LIPUS and HG + LIPIS + 3MA groups; one-way ANOVA. The scale bar was set to 200 μm (*n* = 3). (**C**–**E**) Results of tube formation assay in HG + LIPUS and HG + LIPIS + 3MA groups, quantified with (**D**) the number of junctions, and (**E**) total length; one-way ANOVA. The scale bar was set to 200 μm (*n* = 3). (**F**,**G**) Fluorescence images and statistical analysis of EdU-positive cells in HUVECs in HG + LIPUS and HG + LIPIS + 3MA groups; one-way ANOVA. The scale bar was set to 100 μm (*n* = 3). ns, not significant; * *p* < 0.05, ** *p* < 0.01, *** *p* < 0.001. Error bars represent SD of the mean.

3-Methyladenine (3-MA), a widely used autophagy inhibitor that also suppresses mitophagy, was employed to further investigate the mechanism underlying the therapeutic effect of LIPUS. As previously demonstrated, LIPUS ameliorated high glucose-induced endothelial dysfunction. However, when 3-MA was administered concomitantly, the beneficial effects of LIPUS were markedly attenuated. Compared to the results for the HG + LIPUS group, the addition of 3-MA resulted in impaired migratory and tube-forming capacities of endothelial cells, showing no significant difference from the high-glucose group (Figure 6A–E). Consistent with this, the EdU assay revealed that the number of proliferating HUVECs in the 3-MA co-treated group was significantly lower than that in the HG + LIPUS group and comparable to the level observed in the high-glucose Control group (Figure 6F,G). These results are consistent with the possibility that inhibition of mitophagy weakens the effects of LIPUS.

### 3.6. LIPUS Reverses Aortic Injury at the Histological Level

**Figure 7:**
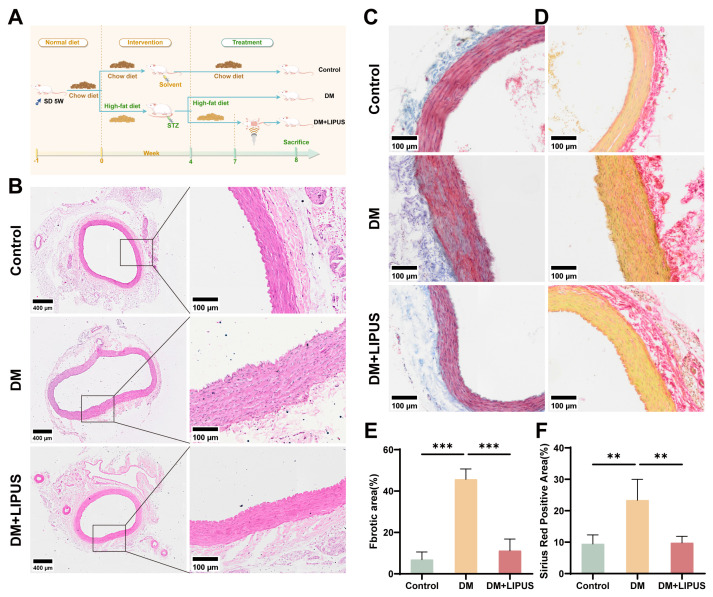
LIPUS reverses aortic pathology changes in diabetic rats. (**A**) Animal experimental protocol, created via FigDraw.com. (**B**) Histological analysis of aortas by H&E staining. Cytoplasm and extracellular matrix appear pink, and nuclei appear blue. The scale bar was set to 100 μm (*n* = 4). (**C**,**E**) Collagen deposition visualized through Masson’s trichrome staining and its quantitative analysis. Collagen fibers appear blue, muscle fibers appear red, and nuclei appear black/dark blue, one-way ANOVA. The scale bar was set to 100 μm (*n* = 4). (**D**,**F**) Collagen content was assessed by Sirius red staining and its quantitative analysis. Collagen fibers appear pink/red, muscle fibers appear yellow, and nuclei appear black/dark blue; one-way ANOVA. The scale bar was set to 100 μm (*n* = 4). ** *p* < 0.01, *** *p* < 0.001. Error bars represent SD of the mean.

Following the 7-day LIPUS treatment, rats from each group were euthanized, and the abdominal aorta was taken for histological staining to further evaluate the impact of hyperglycemia on abdominal aortic pathology. The animal experimental protocol is outlined in Figure 7A. H&E staining displayed that the Control group maintained an intact endothelial layer, characterized by a continuous monolayer of flattened cells and clearly delineated vessel wall structures. In contrast, the DM group displayed varying degrees of endothelial injury, including structural disruption, discontinuity or detachment of endothelial cells, and vascular wall thickening. Notably, the DM + LIPUS group showed reduced abdominal aortic endothelial damage relative to that of the DM group (Figure 7B). Sirius red and Masson staining showed that the Control group had a thin, uniformly structured elastic lamina, whereas the DM group exhibited elastic lamina thickening, fiber disorganization, and substantial collagen deposition. The DM + LIPUS group, however, displayed mild lamina thickening and a slight increase in collagen fibers, indicating a significant reduction in fibrosis relative to that of the DM group (Figure 7C–F).

## 4. Discussion

Sustained hyperglycemia promotes excessive reactive oxygen species generation, contributing to endothelial dysfunction in diabetes [35]. Endothelial dysfunction represents a key pathogenic factor in diabetic vascular complications [36]. LIPUS, a non-invasive physical therapy, has been widely investigated in osteoarticular diseases [37] and cardiovascular disease [38]; however, its potential role in the treatment of diabetic macrovascular complications remains underexplored. Although previous reports have indicated that low-intensity therapeutic ultrasound improves arterial endothelial function in individuals with T2DM [22], researchers only analyzed the findings from a macro perspective and did not elucidate the underlying mechanisms. Following a bioinformatic analysis of RNA-seq data from HUVECs exposed to high glucose, this study employed in vitro and in vivo models to validate the protective effects of LIPUS against hyperglycemia-induced endothelial injury and to delineate the underlying molecular mechanisms.

In the diabetic microenvironment, multiple mechanisms collectively impair cellular migration and tube formation, contributing to delayed wound healing [39,40]. The study confirmed that high glucose markedly suppressed the migration and tube formation capabilities of HUVECs, consistent with bioinformatic predictions and previous findings [41]. After LIPUS intervention, angiogenic potential was restored. Li et al. also demonstrated this finding [42]. In addition, high glucose markedly inhibits endothelial cell proliferation and promotes apoptosis [43,44]. This study also indicated this, and LIPUS effectively reversed these changes. These results demonstrate that LIPUS can counteract high glucose-induced endothelial cell dysfunction.

Mitochondrial dysfunction represents a hallmark of T2DM [45]. Dysfunctional mitochondria overproduce reactive oxygen species, further aggravating mitochondrial damage and establishing a vicious cycle [46]. Mitophagy, a form of selective autophagy, regulates the clearance of damaged mitochondria and is a key mechanism for maintaining mitochondrial quality [8,47]. Mitophagy deficiency is linked to the pathophysiology of T2DM [48]. Meanwhile, bioinformatics analysis suggested that high glucose downregulates pathways related to mitochondria, autophagy, and selective autophagy in HUVECs. Restoring mitophagy has been proposed as a promising therapeutic strategy for T2DM [11]. Notably, therapeutic ultrasound can activate mitophagy. These findings prompted us to further investigate mitophagy as a potential underlying mechanism.

We propose that LIPUS may act through the activation of mitophagy—a notion preliminarily supported by our experimental data. Western blotting and immunofluorescence were used to reveal that high glucose impaired mitophagy, whereas LIPUS treatment may play a role in restoring this process, as evidenced by increased levels of the autophagy marker LC3, decreased levels of the autophagy receptor p62, reduced expression of the mitochondrial outer membrane protein TOM20, and an increased LC3-TOM20 co-localization coefficient. These changes do not occur under physiological conditions. We also observed that co-treatment with 3-MA resulted in a significant attenuation of the therapeutic benefits of LIPUS, further indicating the importance of mitophagy in the action of LIPUS. These results potentially represent a central mechanism of its beneficial effect. Future studies are still needed to detect mitophagy using more direct tools such as mt-Keima [49] and transmission electron microscopy. We suspect that the mechanical stress imposed by LIPUS may be perceived by cells as a physical stimulus that is transduced into biochemical signals, activating mitophagy and alleviating endothelial cell dysfunction resulting from high glucose. Nevertheless, the exact signaling pathway through which LIPUS triggers mitophagy requires further exploration.

In the in vivo experiment, considering that estrogen can enhance insulin sensitivity and delay the onset of diabetes [50], male SD rats were used to establish the T2DM model, with a focus on aortic endothelial integrity and fibrosis. Previous studies have reported increased aortic collagen deposition in T2DM mice [51]. Fibroblasts, as the primary source of the extracellular matrix, play a central role in fibrosis in multiple organs [52,53]. GSEA and GO analysis results suggested that high glucose positively regulates pathways related to fibroblast proliferation. Histological staining results showed arterial wall thickening, loss of endothelial layer integrity, elevated collagen deposition, and pronounced fibrosis in the DM group. After LIPUS treatment, the above-mentioned pathological changes were significantly ameliorated. These results not only confirm the protective role of LIPUS but also extend its demonstrated efficacy from the cellular level to tissue and organ levels. Kajikawa et al. [21] further conducted a human clinical trial, and the results showed that LIPUS significantly alleviated clinical symptoms, controlled inflammation, optimized perfusion parameters, and restored vascular function in patients with atherosclerotic peripheral arterial disease. These findings provide strong support for the subsequent clinical translation of LIPUS therapy in vascular diseases.

The bioinformatics analysis in this study was limited by a small sample size. Future studies should utilize larger cohorts and transcriptomic screening to identify key targets of LIPUS and to elucidate the upstream signaling pathways through which LIPUS activates mitophagy in endothelial cells. Notably, prior evidence indicates that LIPUS may also promote endothelial cell apoptosis and inhibit angiogenesis under certain conditions [54]. The biological effects of LIPUS are parameter-dependent. Therefore, the selection of appropriate treatment parameters is critical. In this work, a single set of parameters derived from previous literature was employed [24]. In the future, comprehensive in vivo and in vitro studies will be conducted to systematically optimize parameters such as intensity, frequency, duty cycle, and treatment duration and to establish the optimal treatment protocol. In addition, the actual acoustic intensity at the target site should be measured, and acoustic calibration accounting for differences in tissue attenuation should be performed to refine the parameters. Furthermore, as a physical intervention, the long-term safety of LIPUS requires further thorough evaluation.

## 5. Conclusions

In summary, this work shows that LIPUS mitigates high glucose-induced endothelial dysfunction in vitro—effects potentially mediated through the activation of mitophagy. In diabetic rats, LIPUS treatment preserved aortic architecture and reduced collagen fiber deposition. These results provide experimental and theoretical support for LIPUS as a non-invasive strategy to counter diabetic macrovascular complications, highlighting its translational potential. However, the upstream mechanism by which LIPUS protects the vascular endothelium through the regulation of mitophagy remains to be further elucidated.

## Data Availability

The original contributions presented in this study are included in the article. Further inquiries can be directed to the corresponding author.

## Supplementary Materials

The following supporting information can be downloaded at: https://www.mdpi.com/article/10.3390/metabo16050329/s1, Figure S1: LIPUS does not activate mitophagy in normal HUVECs.

## Abbreviations

The following abbreviations are used in this manuscript:

LIPUS	Low-intensity pulsed ultrasound
T2DM	Type 2 diabetes mellitus
HUVECs	Human umbilical vein endothelial cells
3-MA	3-Methyladenine
STZ	Streptozotocin
DEGs	Differentially expressed genes
KEGG	Kyoto Encyclopedia of Genes and Genomes
GO	Gene Ontology
GSEA	Gene Set Enrichment Analysis
BP	Biological process
CC	Cellular component
