# Supplementary material for: Regulation of Mitophagy by Low-Intensity Pulsed Ultrasound Attenuates Endothelial Dysfunction
Source: Metabolites. 2026 May 15;16(5):329. doi: 10.3390/metabo16050329 (PMC13208175; doi:10.3390/metabo16050329)
Supplement: Supplementary file 1 [file metabolites-16-00329-s001.zip › Supplementary material.pdf]

Regulation of Mitophagy by Low-intensity Pulsed Ultrasound Attenuates Endothelial Dysfunction

Yucong Shi [1,2], Baotian Zhao [1], Yuhong Wei [1], Dongxu Lu [1,3], Haixia Liu [4] and Yinzhu Chu [1,*]

[1] The First Clinical Medical College of Harbin Medical University, Harbin 150001, China; 2023020734@hrbmu.edu.cn (Y.S.); 2024021131@hrbmu.edu.cn (B.Z.); 2023020763@hrbmu.edu.cn (Y.W.); ludongxu@hrbmu.edu.cn (D.L.)
[2] NHC Key Laboratory of Cell Transplantation, Harbin 150001, China
[3] Heilongjiang Provincial Key Laboratory of Hepatosplenic Surgery, Harbin 150001, China
[4] The Third Clinical College of Harbin Medical University, Harbin 150081, China; 900116@hrbmu.edu.cn (H.L.)
* Correspondence: 811056@hrbmu.edu.cn

Supplementary Material

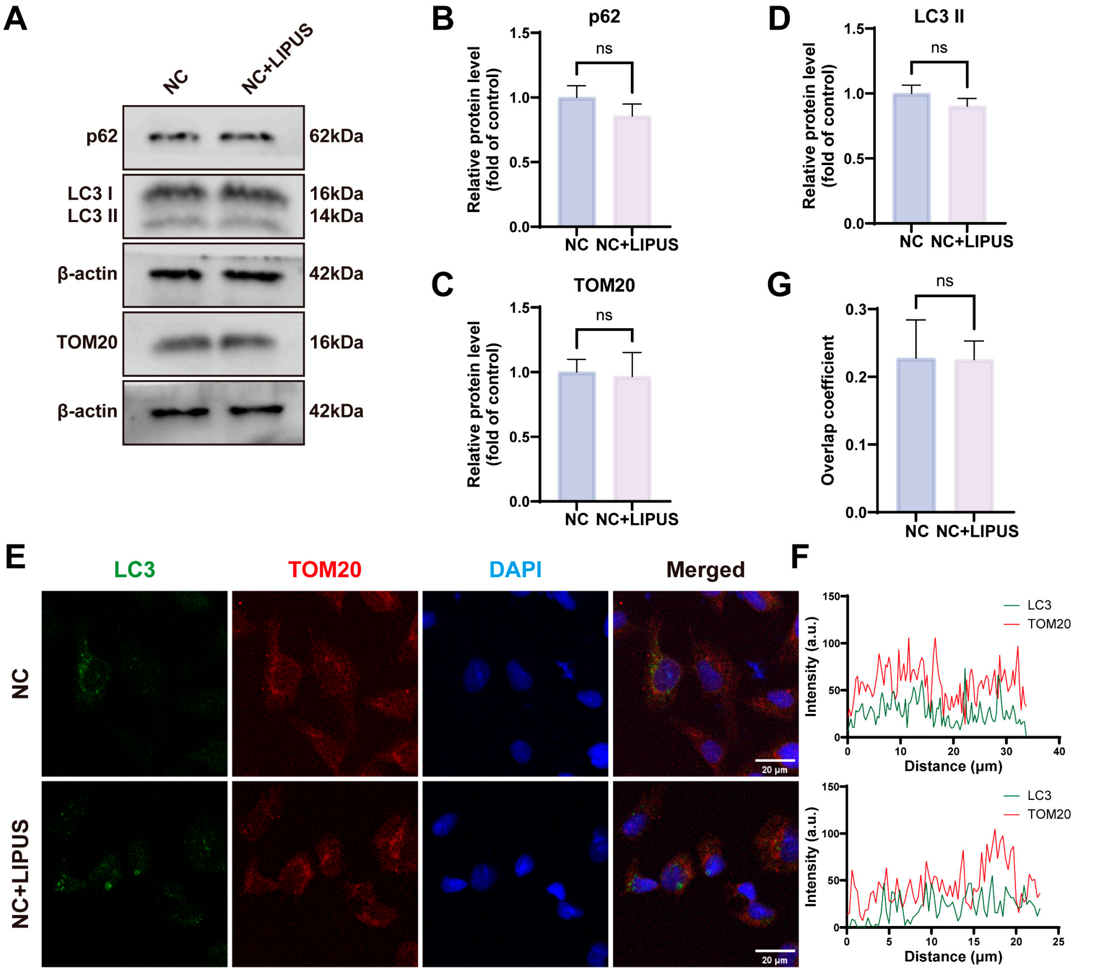

Supplementary Figure S1. LIPUS does not activate mitophagy in normal HUVECs (A-D) Representative protein bands and quantitative statistical results of p62, LC3, and TOM20 protein levels. Two-tailed Student's t-test. (n=3) (E) Immunofluorescence co-staining of LC3 (green), TOM20 (red), and DAPI (blue). The scale bar was set to 20μm. (n=3) (F) Fluorescence intensity distribution profiles of LC3 and TOM20. Green represents LC3, red represents TOM20. (G) Quantification of LC3-TOM20 co-localization. Co-localization was quantified using the Overlap coefficient calculated with ImageJ (Colocalization Finder plugin). A total of 18-21 cells per group were analyzed. Two-tailed Student's t-test. (n=3) ns, not significant. Error bars represent SD of the mean.
