# Supplementary material for: Regulation of Mitophagy by Low-Intensity Pulsed Ultrasound Attenuates Endothelial Dysfunction
Source: Metabolites. 2026 May 15;16(5):329. doi: 10.3390/metabo16050329 (PMC13208175; doi:10.3390/metabo16050329)

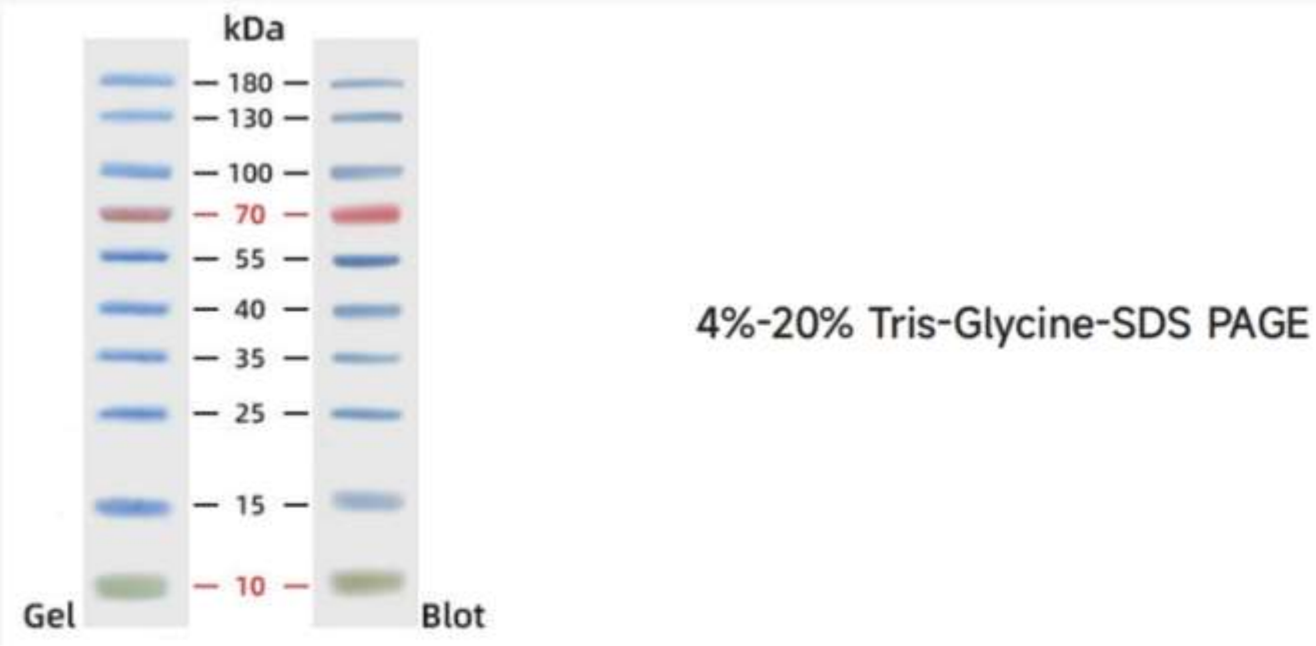

Tricolor pre-stained protein Marker (10 kDa~180kDa)
Epizyme (Shanghai, China)
NO.: WJ107
12.5% Tris-Glycine-SDS PAGE

We would like to provide a sincere explanation regarding the Western blot raw data. In a few membranes, the 180 kDa molecular weight marker is not visible. This is likely because the 12.5% SDS-PAGE used compressed the separation of higher molecular weight proteins, making the marker band difficult to distinguish. Alternatively, given that our target protein has a maximum molecular weight of 62 kDa, a shorter transfer time may have been used, which could have prevented the complete transfer of higher molecular weight proteins (including the 180 kDa marker) onto the membrane.

Figure 4E

①

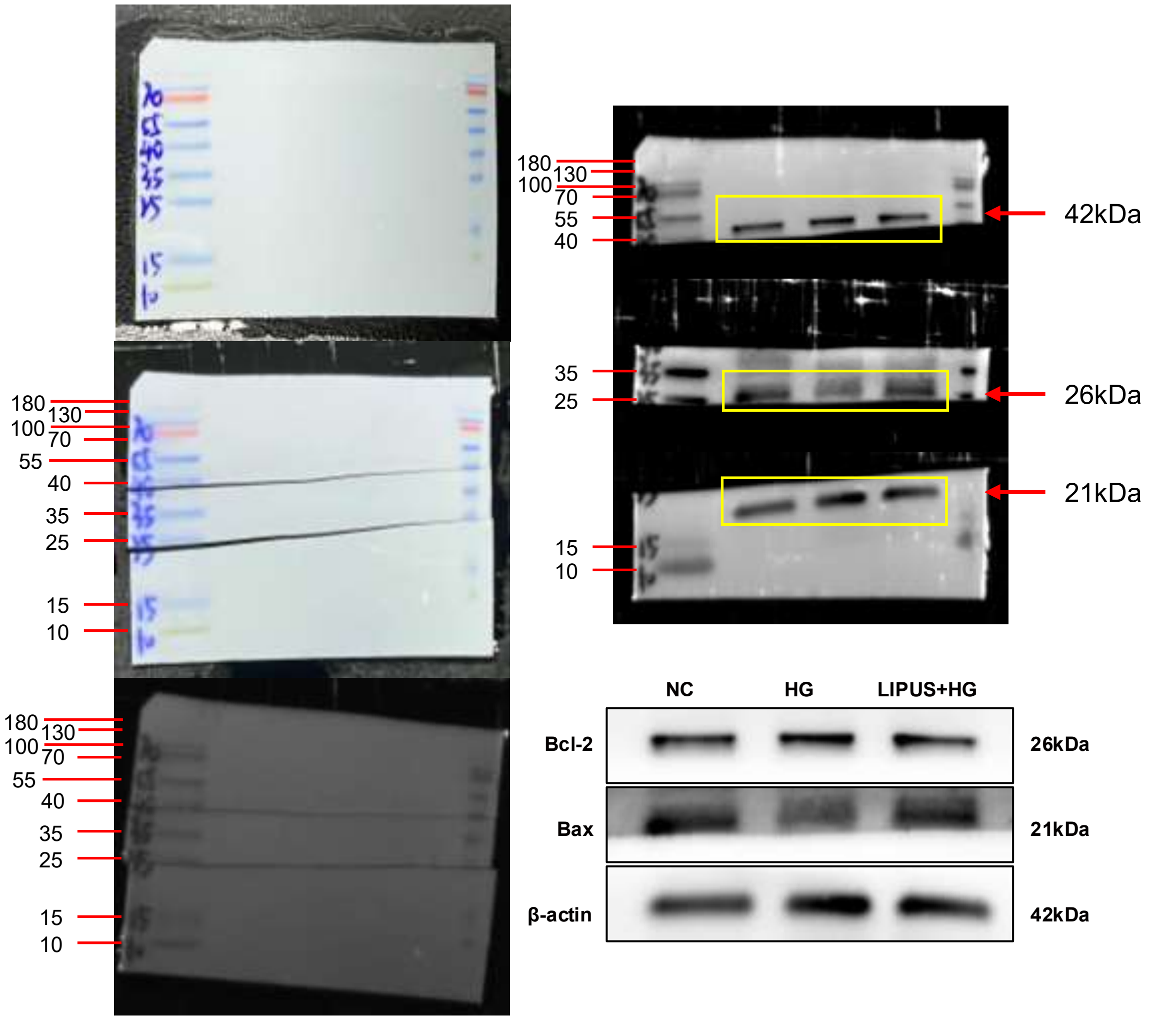

② (Representative image)

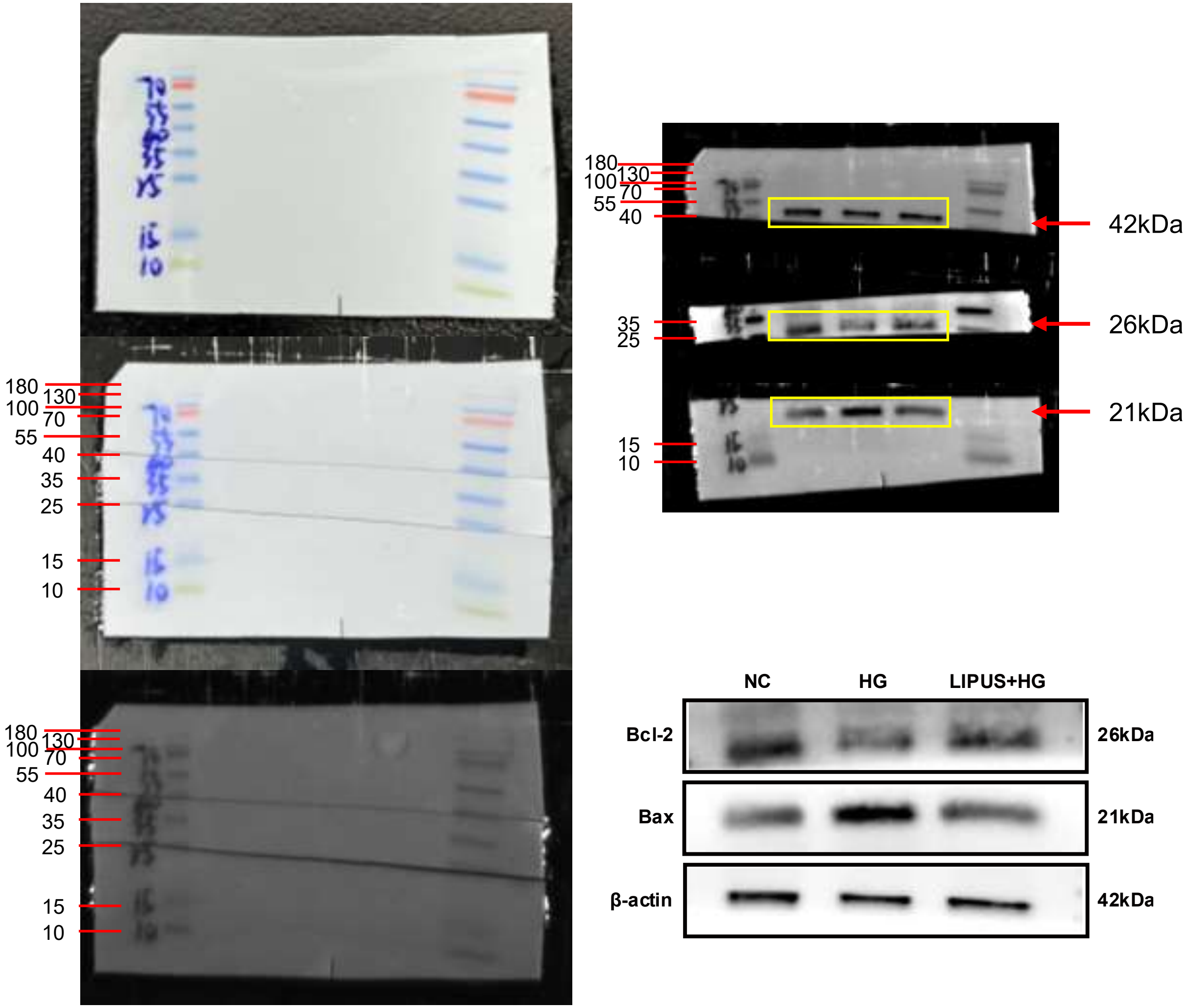

③

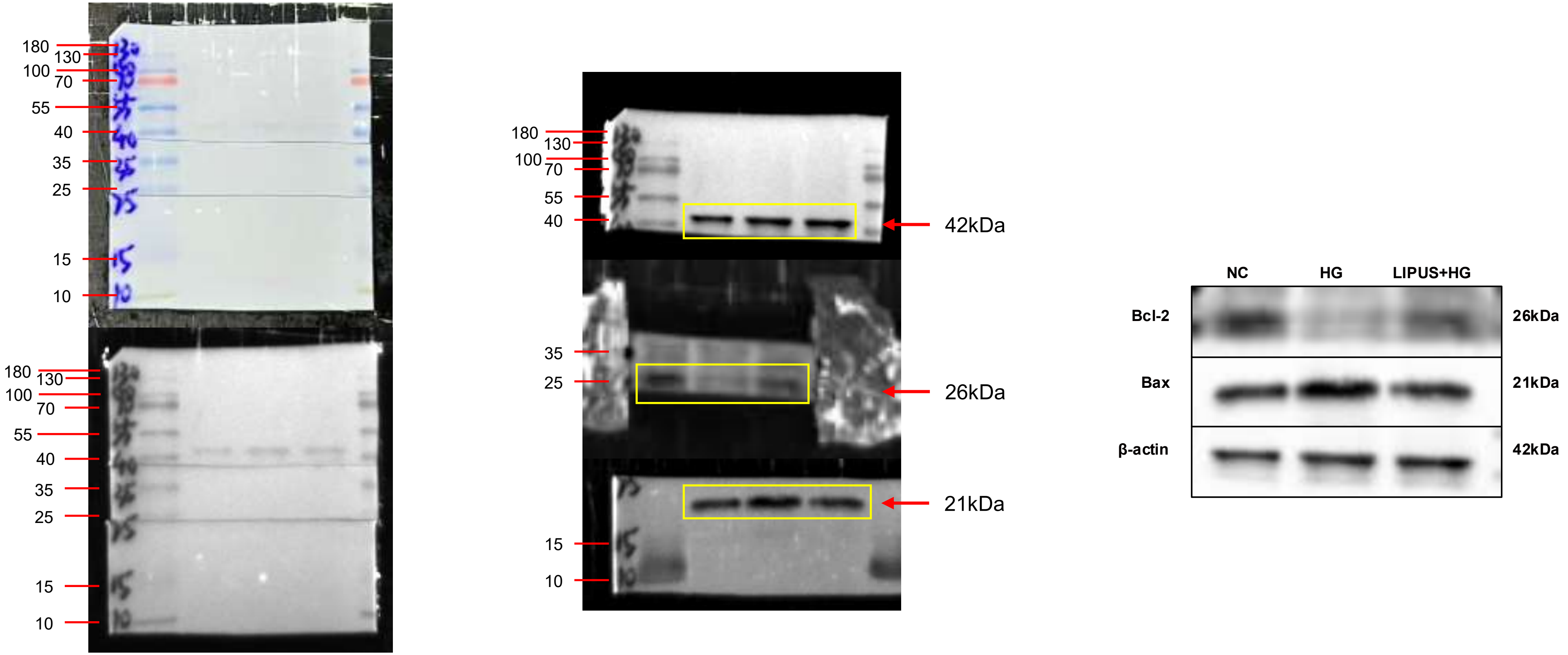

- We sincerely apologize that the molecular weight markers for the second membrane were covered with aluminum foil in Figure 4③ during chemiluminescent development.
- Given that the molecular weight marker was intensely overexposed while the target protein bands were faint, a strategic decision was made to physically shield the marker lanes with aluminum foil during chemiluminescent development. This allowed for a prolonged exposure time to optimally visualize the target protein without saturating the marker signals.

Figure 5A

①+②p62

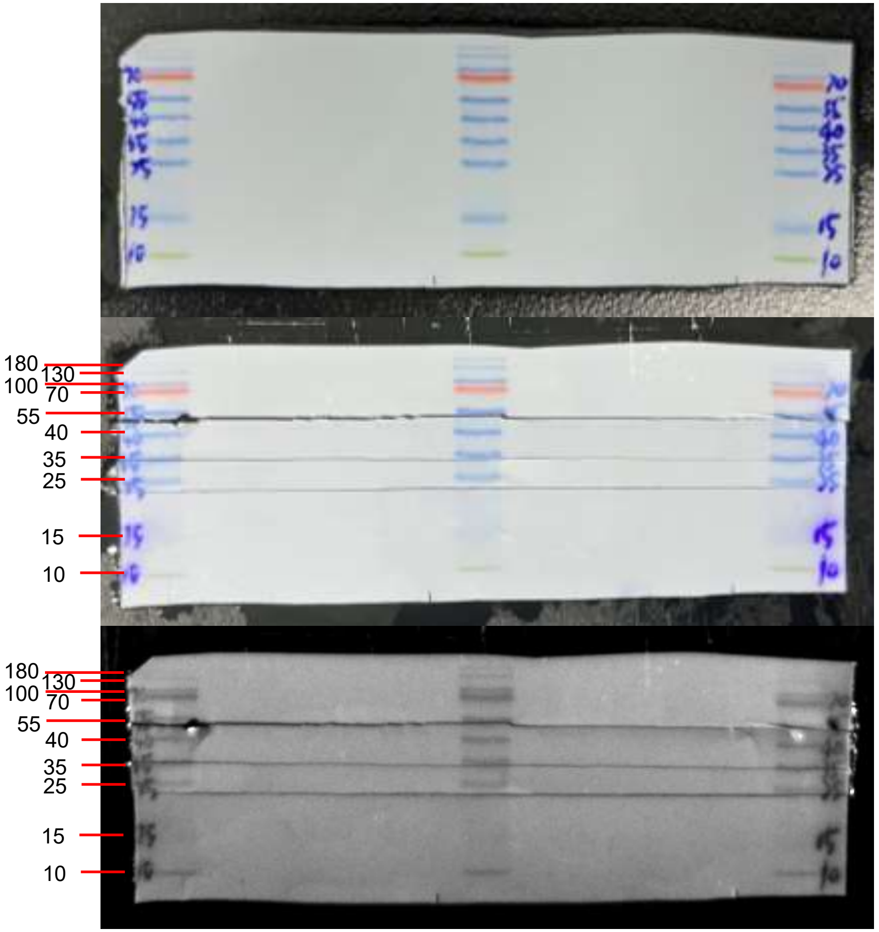

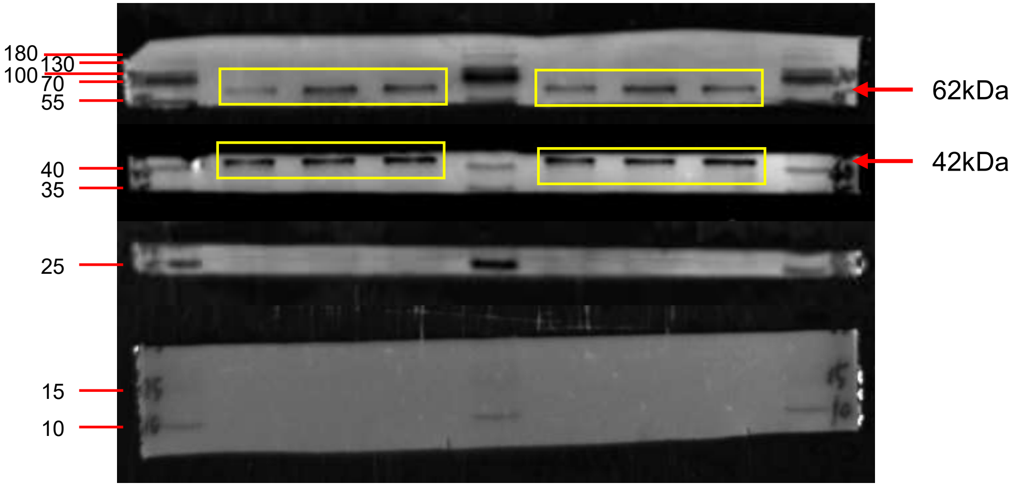

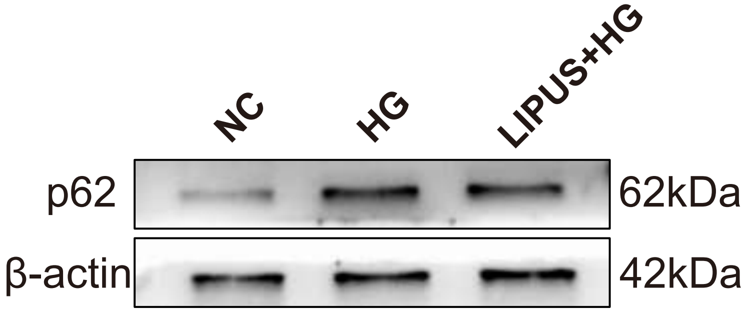

(Representative image)

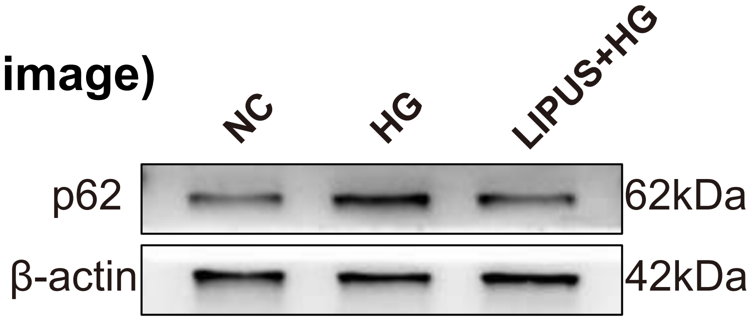

③p62

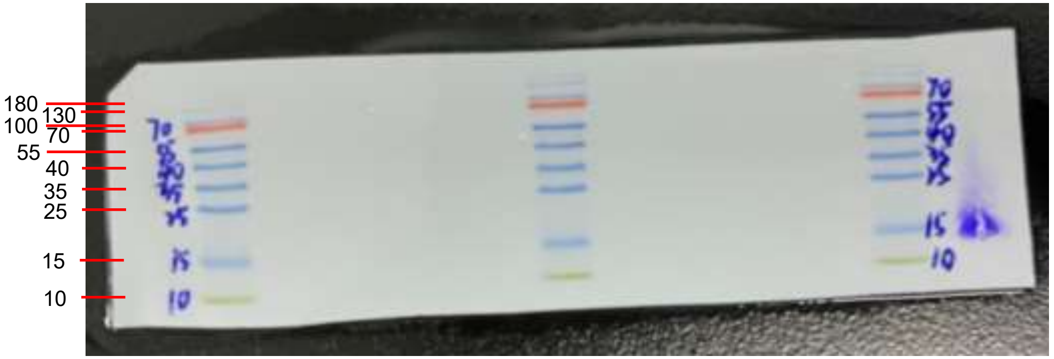

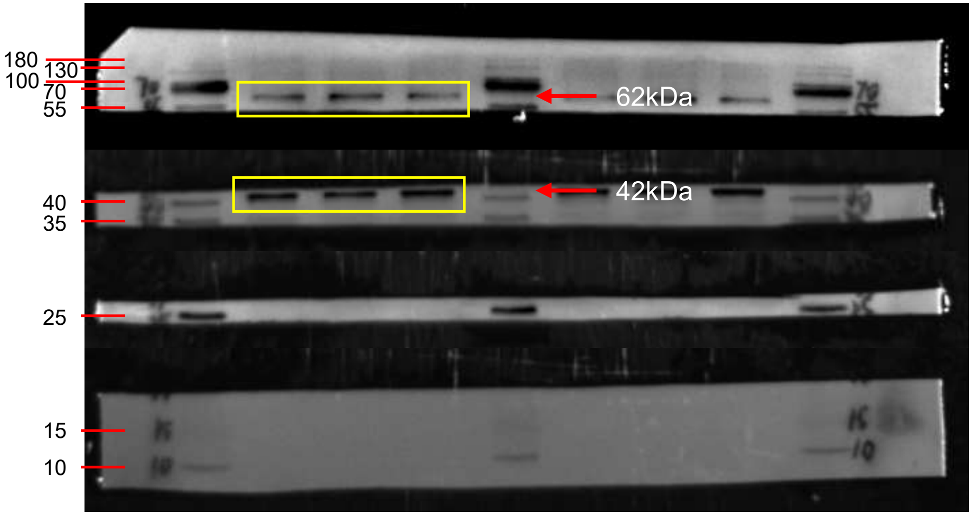

- Only the left half of this blot was used.
- We regretfully admit that we inadvertently failed to capture an image of the blot after cropping and assembly. Fortunately, we had marked the membrane with a ballpoint pen for reference.

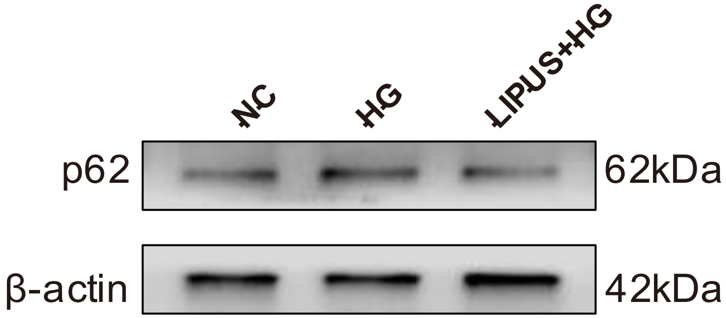

Figure 5A

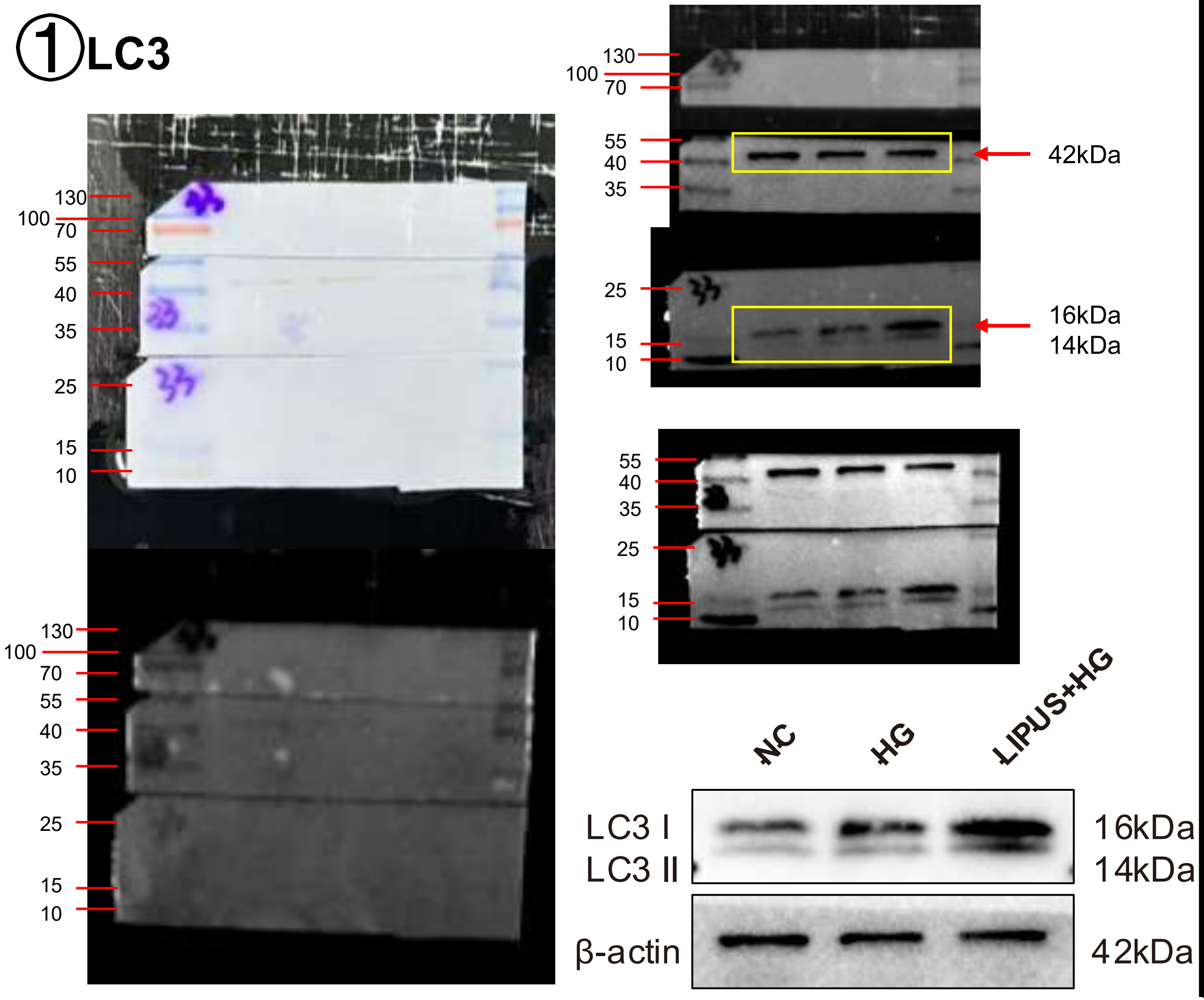

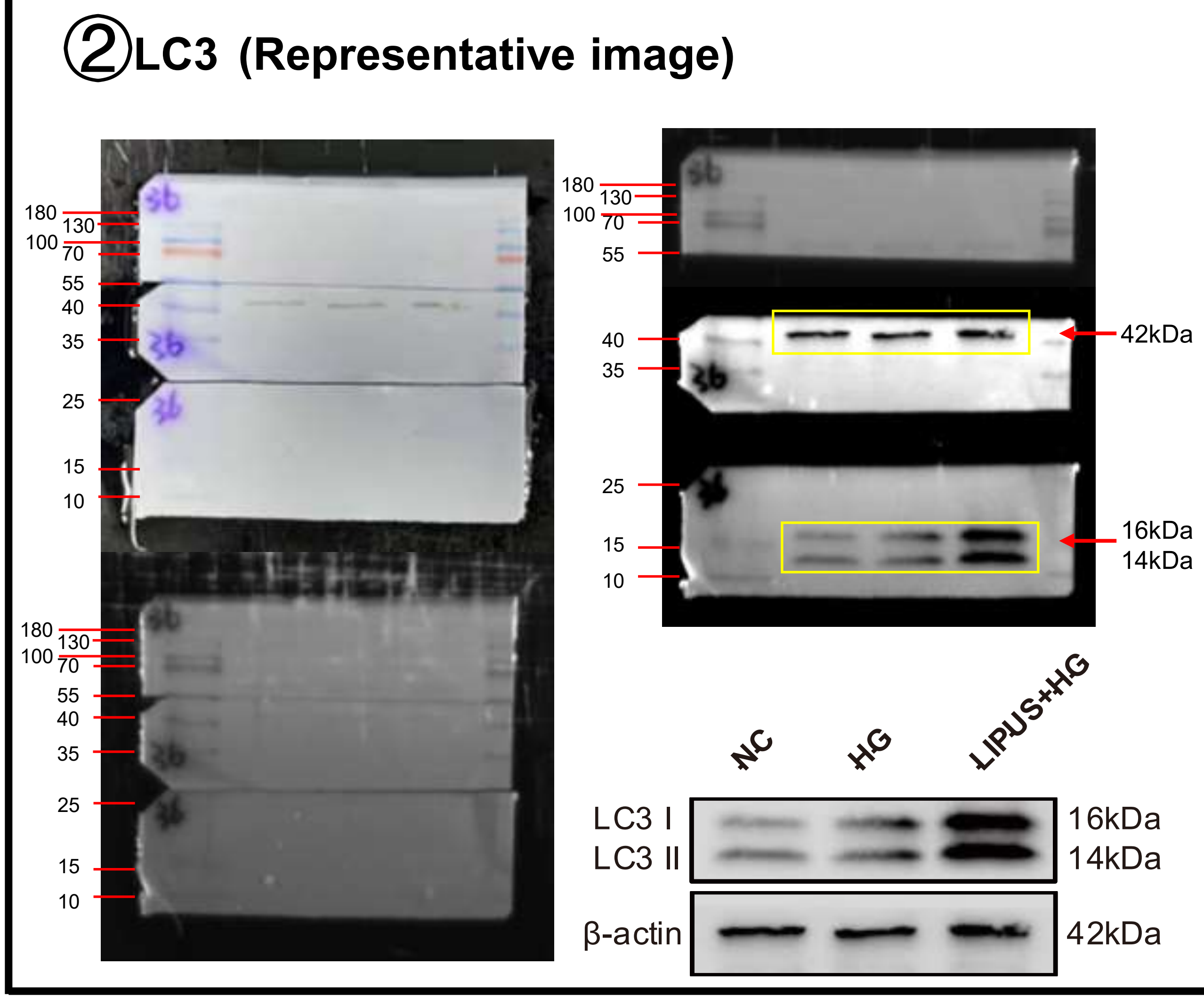

- We would like to explain the inconsistency in the length of the third membrane compared to the other two in Figure 5C ①.
- This variation occurred because, due to the low molecular weight of the target protein, the gel was trimmed into sections and transferred separately to achieve optimal transfer efficiency. Fortunately, the membranes were clearly marked by “33”, confirming that all sections originated from the same original gel.

③LC3

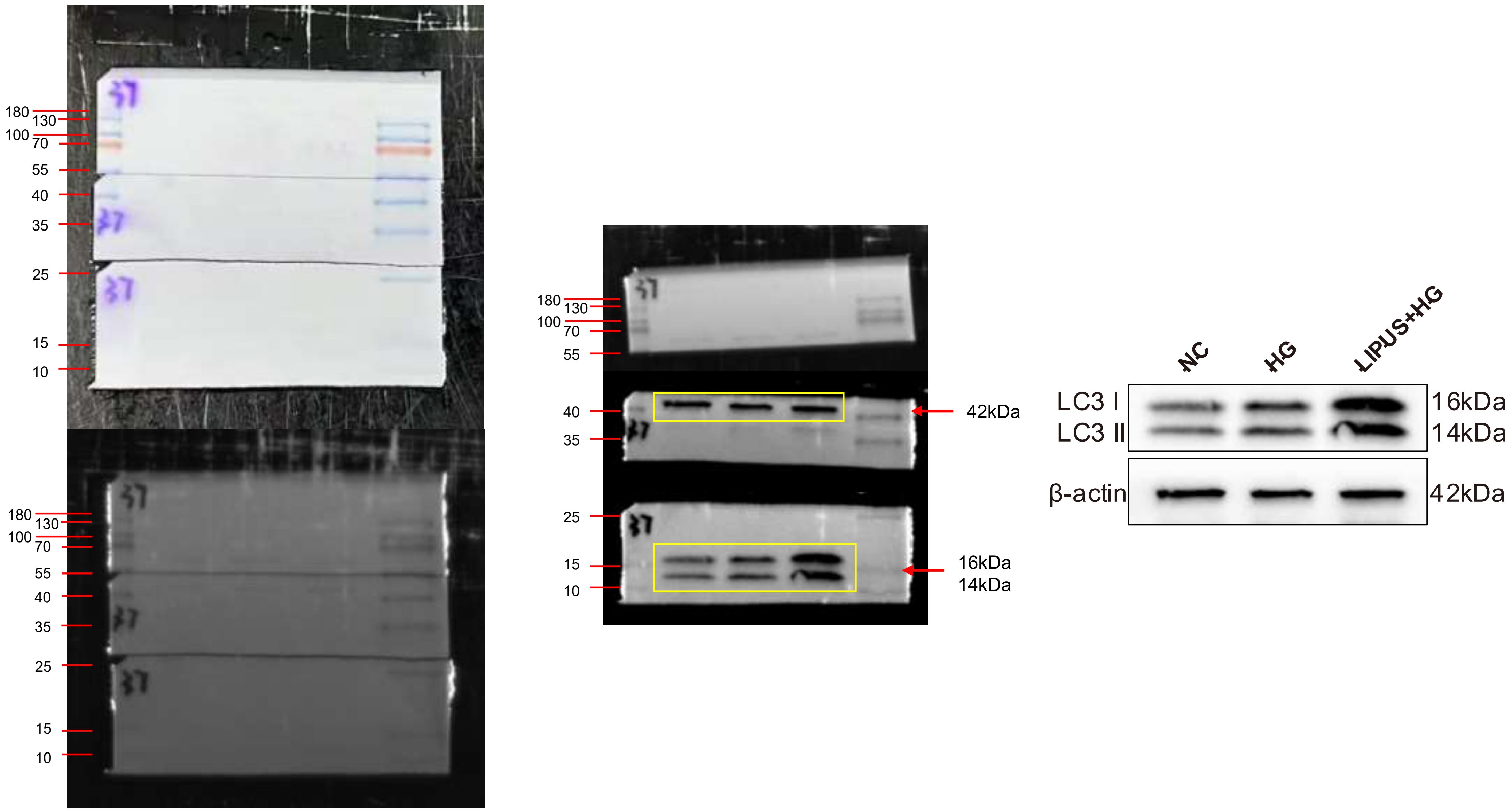

180
130
100
70
55
40
35
25
15
10
42kDa
16kDa
14kDa
NC
HG
LIPUS+HG
LC3 I
LC3 II
β-actin
16kDa
14kDa
42kDa

Figure 5A

①TOM20 (Representative image)

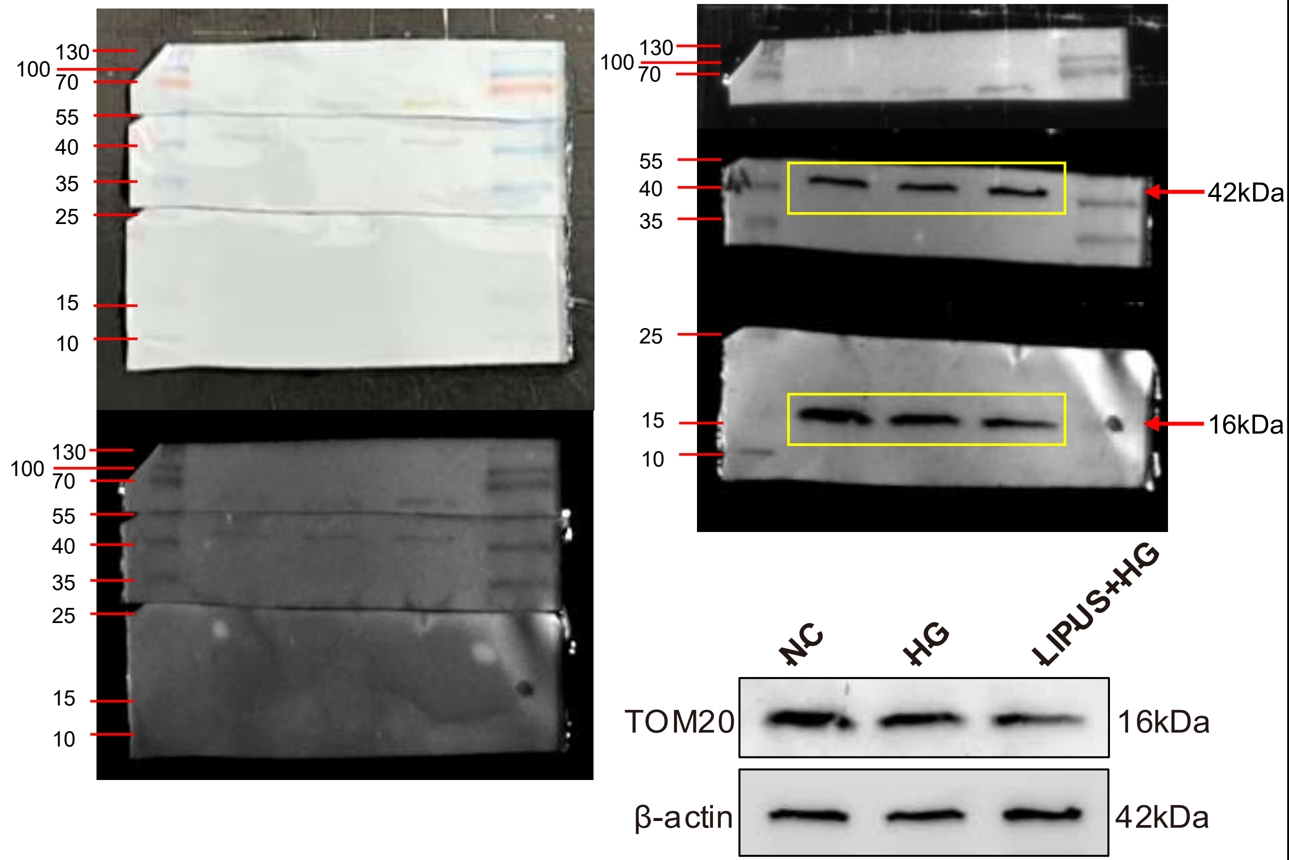

②TOM20

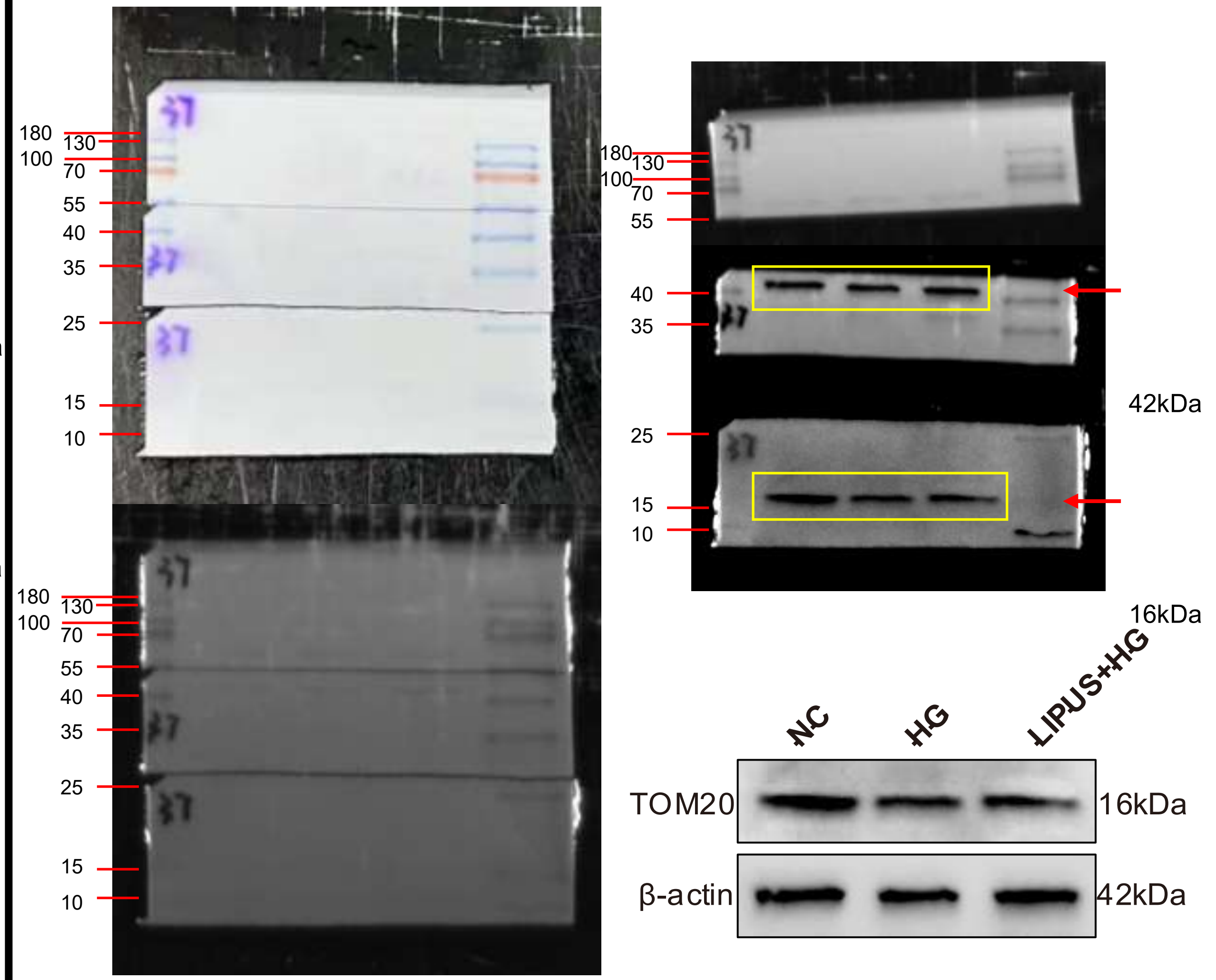

- We sincerely apologize that the molecular weight markers for the third membrane are no longer clearly visible in Figure 5E ①.
- This occurred because we performed a stripping procedure on that portion of the membrane to re-probe it for another target. For reasons that are not entirely clear to us, the original marker signals were significantly diminished during this process. However, it is still evident from the membrane's remaining features that all lanes shown are from the same original membrane.

③TOM20

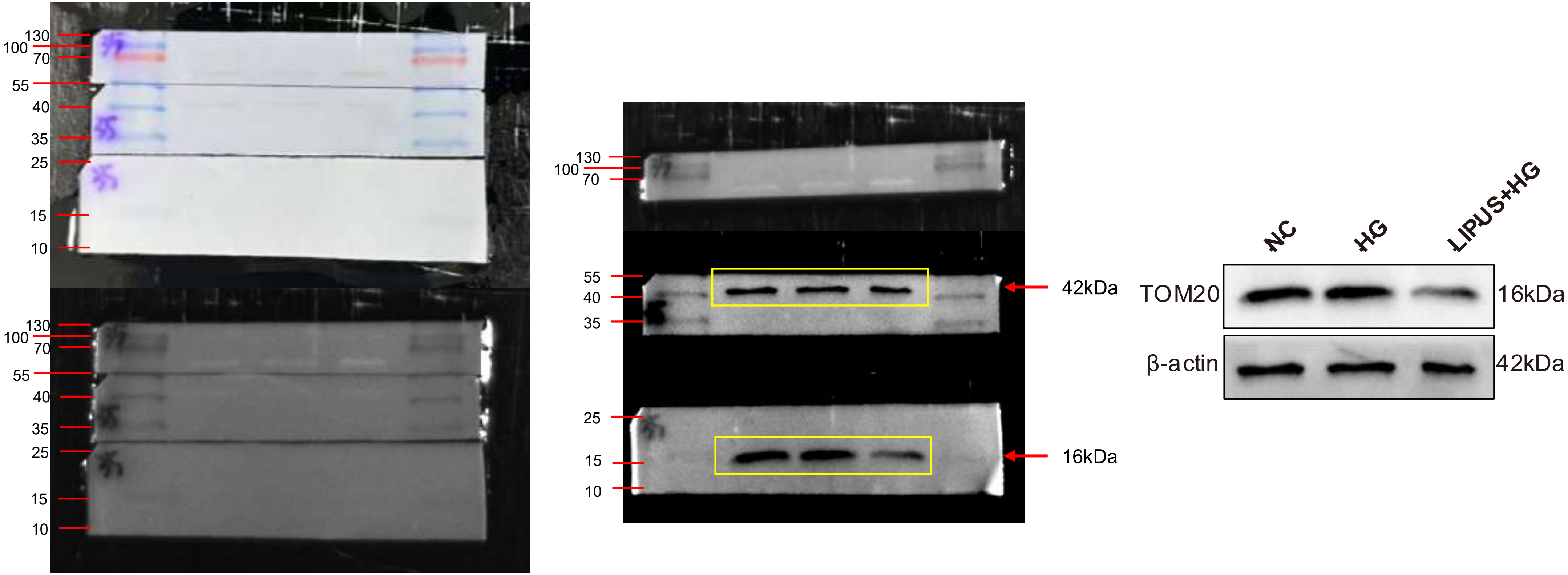

130
100
70
55
40
35
25
15
10
130
100
70
55
40
35
25
15
10
130
100
70
55
40
35
25
15
10
42kDa
16kDa
NC
HG
LIPUS+HG
TOM20
16kDa
β-actin
42kDa

Figure S1A

①p62

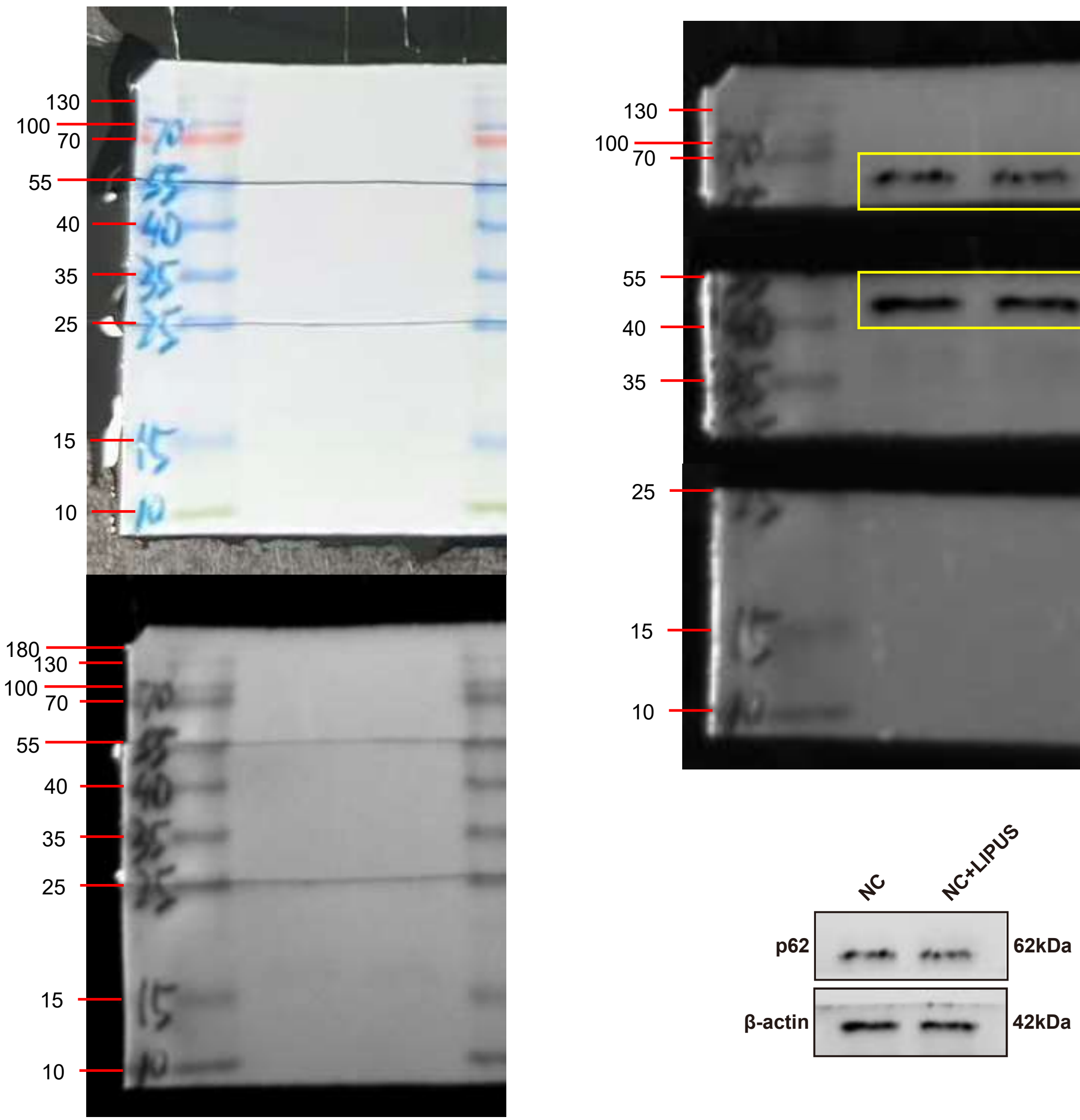

②p62+LC3 (Representative image)

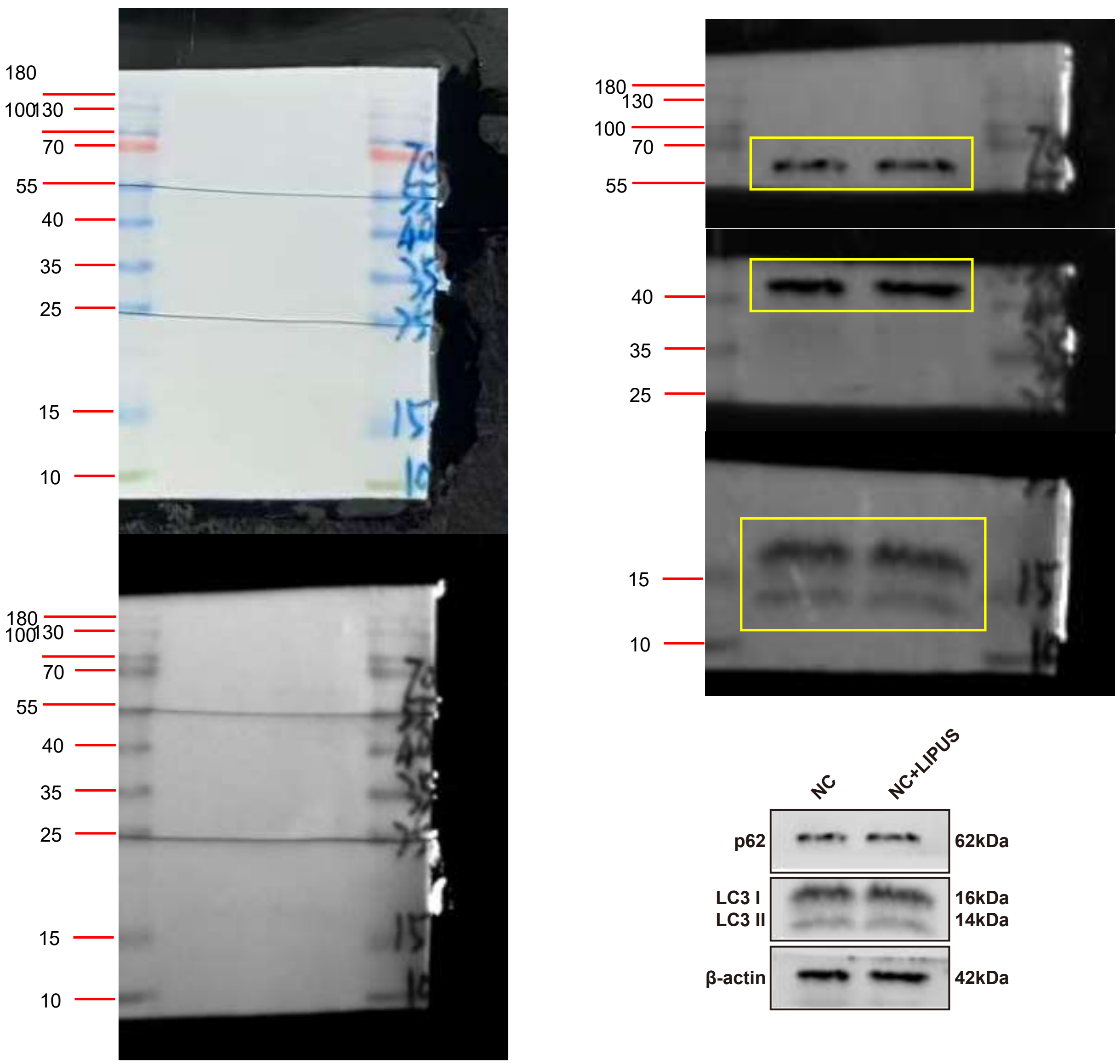

③p62+LC3

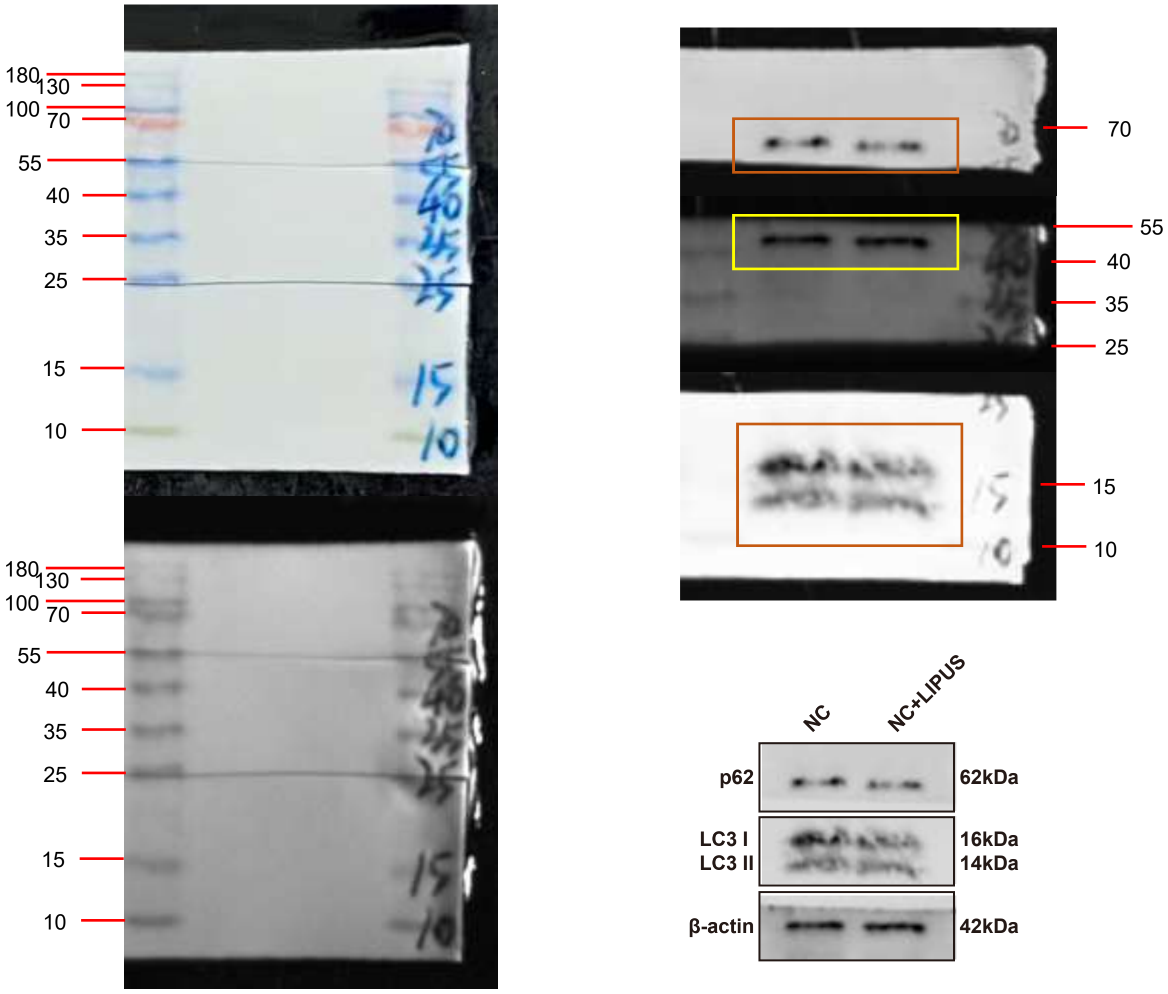

④LC3

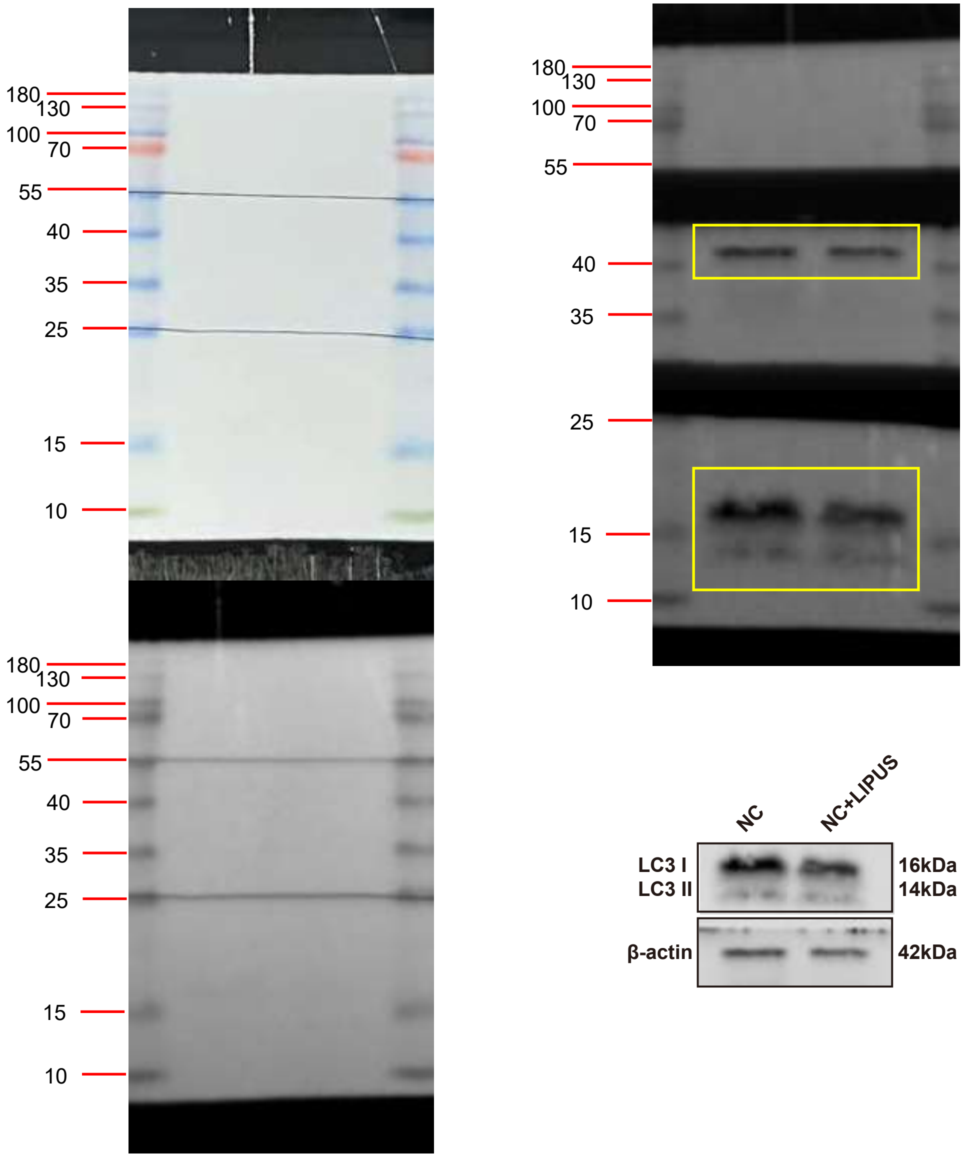

Figure S1A

①TOM20 (Representative image)

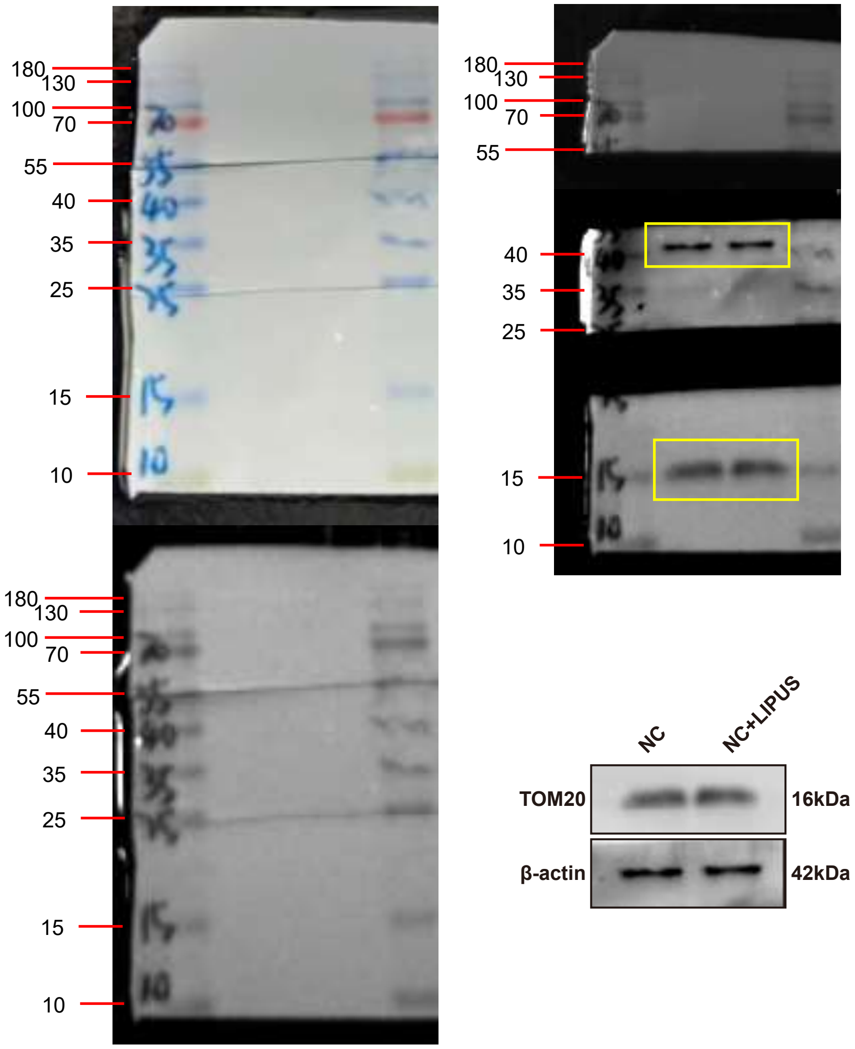

②+③TOM20

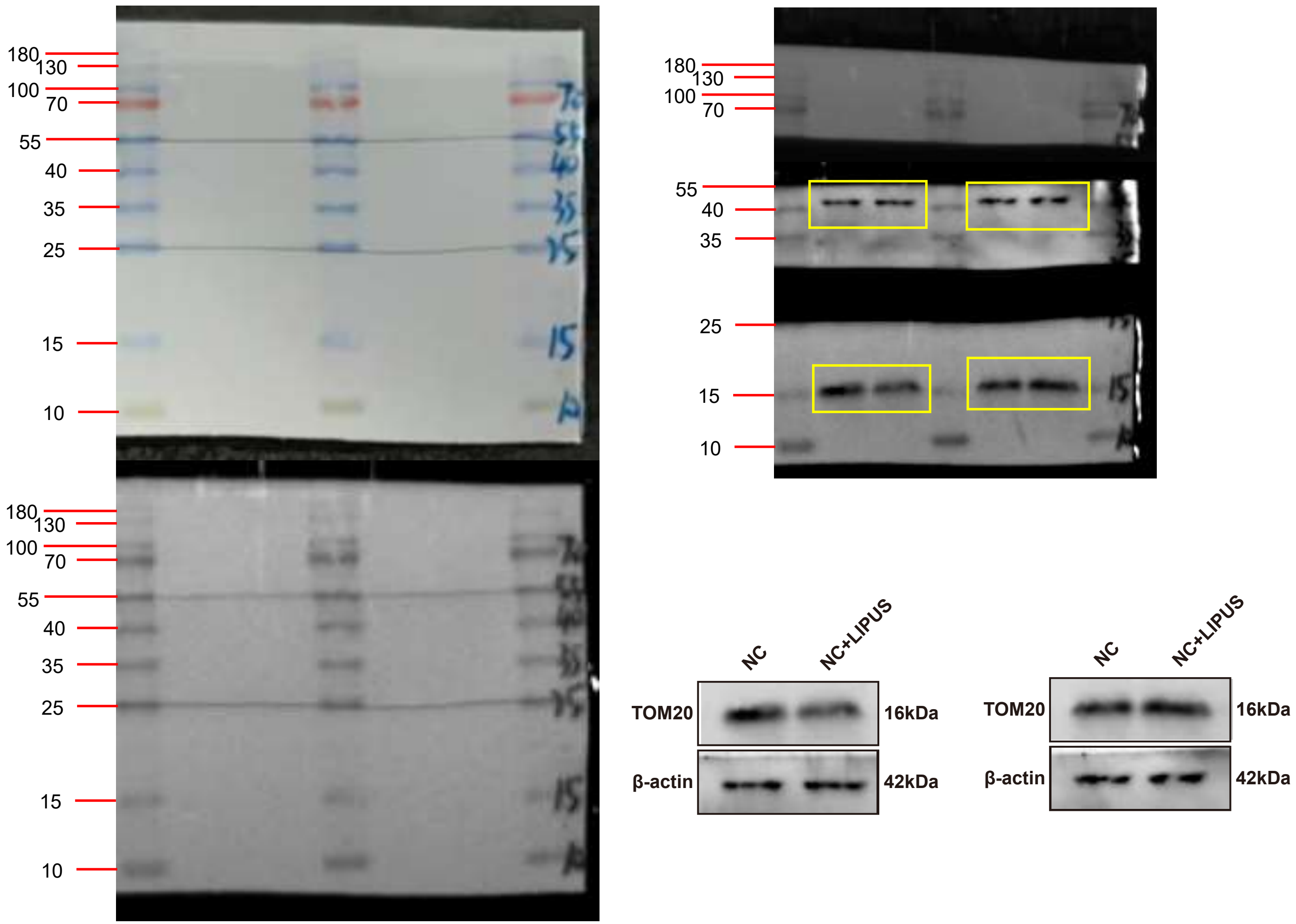

Supplement: Supplementary file 1 [file metabolites-16-00329-s001.zip › Western blot raw data.pdf]
